# Dehydration rapidly induces expression of *NCED* genes from a single subclade in diverse eudicots

**DOI:** 10.1007/s00425-025-04626-z

**Published:** 2025-01-28

**Authors:** Hanh M. Vo, Michael A. Charleston, Timothy J. Brodribb, Frances C. Sussmilch

**Affiliations:** School of Natural Sciences, University of Tasmania, Private Bag 55, Hobart, TAS 7001 Australia

**Keywords:** Dehydration response, Dicot, Evolution, Low humidity, *Nine-*cis*-Epoxycarotenoid Dioxygenase* (*NCED*)

## Abstract

**Main conclusion:**

A gene within a single subclade of *NCED* genes is triggered in response to both, short- and long-term dehydration treatments, in three model dicot species.

**Abstract:**

During dehydration, some plants can rapidly synthesise the stress hormone abscisic acid (ABA) in leaves within 20 min, triggering the closure of stomata and limiting further water loss. This response is associated with significant transcriptional upregulation of *Nine-*cis*-Epoxycarotenoid Dioxygenase* (*NCED*) genes, which encode the enzyme considered to be rate-limiting in ABA biosynthesis. However, most embryophyte species possess multiple *NCED* genes, and it is not currently known whether there is any phylogenetic pattern to which *NCED* genes are involved in this response. We tested transcriptional responses to dehydration for all *NCED* genes present in three diverse eudicot species—*Arabidopsis thaliana* (Arabidopsis), pea and tomato—over both the timeframe of stomatal responses (< 20 min) and in response to sustained dehydration (hours). We found that there is a single *NCED* gene per species, *AtNCED3, PsNCED2,* and *SlNCED1*, respectively, that is rapidly upregulated by dehydration. Using a null mutant*,* we confirmed that the rapidly responsive gene identified in Arabidopsis is important for physiological responses to a sudden drop in humidity. Analysis of the relationships and the evolutionary history of *NCED* genes using available sequence data from diverse land plant species revealed that the identified genes in each species all belong to the same subclade within the gene family, suggesting a conserved role for this subclade in rapid dehydration responses in eudicots. These findings enable future phylogenetically-informed prediction of genes of interest for rapid dehydration responses within this important multigene family in eudicot species.

**Supplementary Information:**

The online version contains supplementary material available at 10.1007/s00425-025-04626-z.

## Introduction

The evolution of adjustable stomatal pores, capable of closing to prevent excessive water loss, and reopening to allow carbon dioxide (CO_2_) acquisition in tissues protected by a waxy cuticle, was a major step in land plant evolution. In terrestrial environments, conditions including atmospheric humidity can change suddenly during the day. Low atmospheric humidity drives increased transpiration and plant water loss through stomata. Dehydration stress affects plant cell turgor and/or cell wall rigidity, which triggers abscisic acid (ABA) biosynthesis in angiosperm species (Pierce and Raschke 1980, 1981; McAdam and Brodribb 2016; Sack et al. 2018; Bacete et al. 2022). ABA activates stomatal closure, limiting further water loss within timeframes as short as 20 min (Mittelheuser and Van Steveninck 1969; Kriedemann et al. 1972; McAdam et al. 2016). This response is associated with rapid transcriptional upregulation of *Nine-*cis*-Epoxycarotenoid Dioxygenase* (*NCED*) genes (McAdam et al. 2016), which encode the enzyme considered to be rate-limiting in ABA biosynthesis, catalysing the cleavage of 9-*cis*-violaxanthin/9-*cis*-neoxanthin to produce xanthoxin (Tan et al. 1997; Qin and Zeevaart 1999). Other mechanisms for increasing ABA levels, including ABA release from ABA-glucosyl ester (ABA-GE) storage (Dietz et al. 2000; Lee et al. 2006; Xu et al. 2012; Georgopoulou and Milborrow 2012; Mercado-Reyes et al. 2024) or decreased ABA catabolism (Kushiro et al. 2004; Saito et al. 2004; Umezawa et al. 2006; Ren et al. 2007), may regulate ABA levels in other circumstances. However, these mechanisms do not appear to play an important role in increasing ABA levels during rapid dehydration responses, as ABA-GE levels have been found to remain similar or increase, while transcription of ABA catabolism genes is upregulated in response to low humidity or altered leaf turgor pressure over these time frames (McAdam et al. 2016; Sussmilch et al. 2017). The importance of de novo ABA biosynthesis in rapid dehydration responses is supported by studies showing that ABA biosynthesis mutants do not show the same rapid increases in ABA levels as wild-type plants, leading to a characteristic ‘wilty’ phenotype in response to low humidity (McAdam et al. 2015, 2016).

Most embryophyte species possess multiple *NCED* genes, which form a land plant-specific clade within the wider carotenoid cleavage dioxygenase (CCD) family (Sussmilch and McAdam 2017; Jiao et al. 2020). There is evidence of some diversification among angiosperm *NCED* genes in terms of expression patterns, tissue-specificity and transcriptional responses, with specific roles including short- and/or long-term dehydration responses, and seed dormancy (Tan et al. 2003; Lefebvre et al. 2006; Frey et al. 2012; Zdunek-Zastocka and Sobczak 2013; Ji et al. 2014; McAdam et al. 2016). However, systematic testing of rapid transcriptional responses to dehydration for all *NCED* genes in a species, over both the timeframe of stomatal responses (< 20 min) and in response to sustained dehydration (hours), is currently lacking.

To test if there is a phylogenetic pattern between *NCED* genes that are rapidly induced by dehydration in eudicots, we examined the change in expression levels after two different dehydration treatments for all *NCED* genes present in three diverse eudicot species: *Arabidopsis thaliana* (Arabidopsis, eurosid—malvid); *Pisum sativum* (pea, eurosid—fabid); *Solanum lycopersicum* (tomato, asterid). We found that there is one *NCED* gene per species that is rapidly upregulated by dehydration within short (minutes) and longer (hours) timeframes. We confirmed the importance for rapid response at a physiological level with mutant analysis. We analysed the relationships and evolutionary history of *NCED* genes more widely in diverse land plants using available sequence data. We found that each rapidly responsive *NCED* gene identified belongs to the same subclade within the gene family, suggesting a conserved role for this subclade in rapid dehydration responses in eudicots.

## Materials and methods

### Plant material and dehydration treatment

*Arabidopsis thaliana* Col-0, *Pisum sativum* cultivar (cv.) Torsdag, and *Solanum lycopersicum* cv. Rheinlands Ruhm plants were grown under short-day condition (8 h light/16 h dark) at 25 °C day/20 °C night and watered daily. For pressurisation experiments, all plants were covered overnight using a plastic bag to ensure full plant turgor at the beginning of the experiment. Leaves were dehydrated in a Scholander pressure chamber (Tyree and Hammel 1972; McAdam and Brodribb 2016) to control decreases in leaf water potential using specific pressure magnitude from mild (0.2 MPa) to severe (1.0 MPa) for 20 min then depressurised slowly and equilibrated for 20 min in a sealed bag at 100% humidity. Samples were collected for quantification of leaf water potential using the pressure chamber and gene expression for all *NCED* genes in the three species using qRT-PCR. To test responses to severe long-term dehydration, plant roots were cut off with the remaining stem and leaves enclosed in bags which were periodically removed to control drying. Water potential and *NCED* gene expression were quantified at key time points.

### Mutant selection and humidity treatment

Col-0 wild-type plants were grown on 0.5 × Murashige and Skoog Basal Salt mixture (MS) agar without any selection media. Seeds for the *nced3-2* mutant were obtained from the Arabidopsis Biological Resource Centre (ABRC; Ohio State University, Ohio, USA; accession number CS412308) and grown on 0.5 × MS agar with sulfadiazine (7.5 mg/L) for mutant screening. Both genotypes were transferred to soil at 10 days old and grown under the conditions specified above. For low humidity treatment, six-week-old plants were covered overnight using a transparent plastic bag, and exposed to a change of humidity from 92.17% ± 0.42% to 35.07% ± 6.01% relative humidity at 22.46 ± 0.66 °C. All experiments were conducted at the same time of day. Leaf water potential was measured using a Scholander pressure chamber (Tyree and Hammel 1972).

### RNA RT-qPCR quantification and statistical analysis

RNA was extracted with the Isolate II RNA Plant Kit (Meridian Bioscience, Cincinatti, Ohio, USA) using the manufacture’s protocol, except for the following modifications: all centrifuge steps were performed at 4 °C, and the kit’s on-membrane DNAse digestion was replaced with off-membrane digestion with RNAse-free DNase I, and precipitation with sodium acetate (pH 8) and ethanol and resuspension in RNAse-free water.

RNA quantification, reverse transcription, and quantitative reverse transcription PCR (qRT-PCR) were conducted as previously described (McAdam et al. 2016; Sussmilch et al. 2017). Transcript levels for each gene of interest were evaluated for three replicates per species/timepoint against housekeeping genes *PsHel, AtMON1* and *SlTIP41* for pea, Arabidopsis, and tomato, respectively. Primer details are available in Table S1. Dunnett posthoc analysis (Dunnett 1955, 1964) was carried out in R v.4.2.3 and RStudio (RStudio Team 2018; R Core Team 2021) using the tidyverse package (Wickham et al. 2019) and results are available in Tables S2 and S3.

## Results and discussion

### A single *NCED* gene in each dicot species is rapidly upregulated by dehydration

**Fig. 1:**
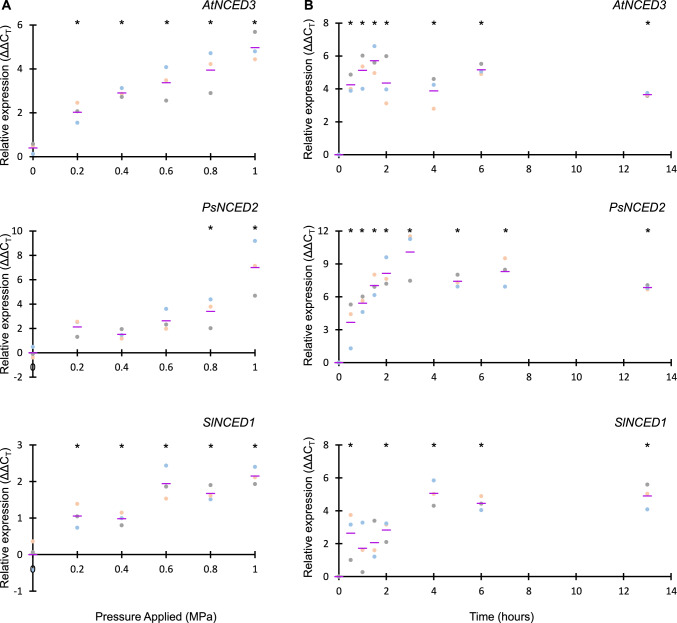
Expression of the *NCED* genes rapidly induced in response to decreased water potential from **a** pressurisation treatment and **b** controlled drying in *Arabidopsis thaliana* (*At*), *Pisum sativum* (pea; *Ps*), and *Solanum lycopersicum* (tomato; *Sl*). Other *NCED* genes showed no rapid increase in expression when exposed to each dehydration stress (Figs. S2 and S3). Mean expression (purple line) and individual biological replicates (*n* = 3, coloured points) relative to control values are displayed, with results of one-way ANOVA with Dunnett’s multiple comparison test indicated (* *P* < 0.05. Full details in Tables S2 and S3)

We first tested the rapid transcriptional response of *NCED* genes to controlled decreases in leaf water potential using different pressure magnitude from mild (0.2 MPa) to severe (1.0 MPa) for Arabidopsis*,* pea, and tomato. Samples were collected for (i) quantification of leaf water potential using the pressure chamber (Fig. S1) and (ii) gene expression for all *NCED* genes in the three species (Fig. 1a; Fig. S2; Table S2). We found that only one *NCED* gene per species was induced within this timeframe (*AtNCED3, PsNCED2, SlNCED1*; Fig. 1a; Fig. S2).

To test responses to severe long-term dehydration, plant roots were cut off with the remaining stem and leaves enclosed in bags, which were periodically removed to control drying. Water potential and *NCED* gene expression were quantified at key time points (Fig. 1b; Figs. S1b and S3). The same *NCED* genes were found to have an increase in response to this dehydration stress over 13 h (*AtNCED3, PsNCED2, SlNCED1*; Fig. 1b; Fig. S3). We confirmed the importance of *AtNCED3* in stabilising water potential rapidly in response to dehydration by exposing the Arabidopsis *nced3-2* mutant (Urano et al. 2009) and wild-type plants to low humidity. We found that mutant plants have significantly lower water potential than wild type within 20 min of low humidity treatment, confirming a physiological role for *AtNCED3* in rapid dehydration response (Fig. S4). These results are further supported by previous studies that show mutants of *AtNCED3* and *SlNCED1* in Arabidopsis and tomato, respectively, maintain low ABA levels, and show higher transpiration rates and higher stomatal conductance compared to wild-type plants during dehydration treatments (Burbidge et al. 1999; Iuchi et al. 2001; Frey et al. 2012; McAdam et al. 2016). All three of the genes—*AtNCED3, SlNCED1* and *PsNCED2*—have previously been shown to have a close temporal relationship between transcriptional upregulation and increased ABA levels (Zdunek-Zastocka and Sobczak 2013; McAdam et al. 2016). A role for *AtNCED5* in sustained dehydration responses has been identified in a previous study, with the *nced3 nced5* double mutant found to show a more severe wilting phenotype than the *nced3* single mutant in response to drought treatment (Frey et al. 2012). However, the *nced5* single mutant shows comparable dehydration responses to wild type (Frey et al. 2012), suggesting that while there may be some redundancy between *NCED5* and *NCED3* during longer dehydration stress, *NCED5* does not play a critical role in rapid dehydration.

### *NCED* genes that respond rapidly to dehydration belong to the same subclade

**Fig. 2:**
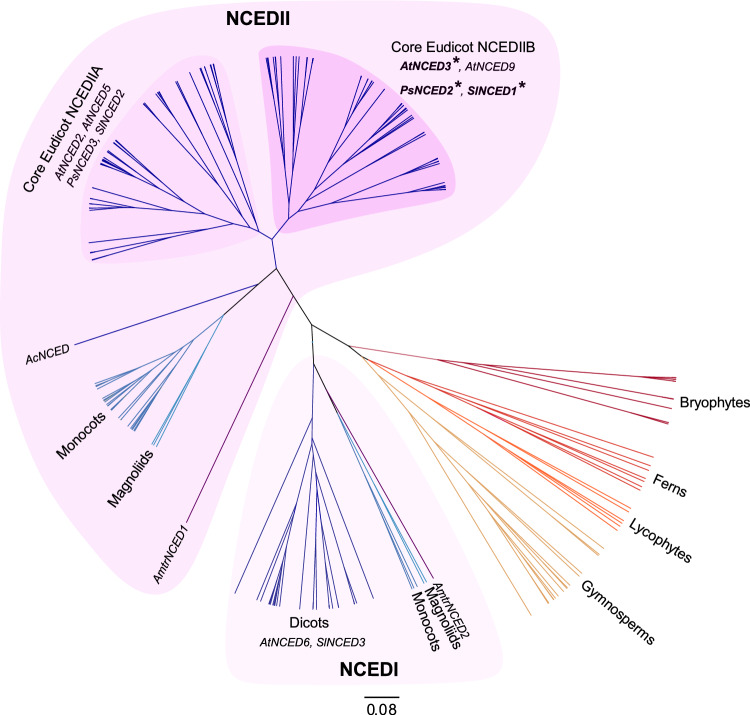
Inferred phylogeny of *NCED* genes in land plants. The full BEAST tree is shown in Fig. S5 using the sequences shown in Table S4. *NCED* genes that were included in gene expression experiments are listed, and those rapidly induced in response to dehydration (Fig. 1) are in bold and are marked with an asterisk (*). Scale bar represents number of nucleotide changes per site

We investigated the phylogenetic relationship of *NCED* genes in major land plant lineages, using available sequence data for 67 diverse land plant species (Fig. 2; Table S4; Fig. S5). The resulting phylogeny reveals separate expansion of the *NCED* gene family in all major land plant lineages except lycophytes; currently, lycophyte genome and transcriptome sequences show a single *NCED* copy per species (Fig. S5). We found two main angiosperm subclades (NCEDI and NCEDII) to be present in diverse species including the “basal” angiosperm *Amborella trichopoda,* which is sister to all other angiosperms (Fig. 2), suggesting that these clades arose from a gene duplication event in shared common ancestor, consistent with the findings of previous analyses using fewer species (Sussmilch and McAdam 2017; Wang et al. 2017). Our analyses further indicate that after the divergence from a common ancestor with *Aquilegia coerulea,* core eudicots underwent another gene duplication in the NCEDII subclade, giving rise to two subclades that we named Core Eudicot NCEDIIA and NCEDIIB. Interestingly, *AtNCED3, PsNCED2* and *SlNCED1* all belong to the Core Eudicot NCEDIIB subclade, suggesting genes in this clade may have a conserved role in dehydration responses, and indicating this as the best subclade to search for candidates for these responses in other eudicot species.

Accordingly, the Core Eudicot NCEDIIB subclade also includes the common bean gene *PvNCED1* (Fig. S5)*,* which has previously been found to show rapid increases in transcript and protein levels, closely correlated with increases in ABA levels, in response to dehydration (Qin and Zeevaart 1999). Arabidopsis has a second gene in the Core Eudicot NCEDIIB subclade, *AtNCED9*, which did not show rapid transcriptional induction in leaves in response to dehydration in our study (Figs. S2 and S3), suggesting some potential loss of this functionality within the clade. Nevertheless, we observed slightly higher expression of *AtNCED9* past the turgor loss point (Figs. S1, S2, and S3), consistent with other studies showing weak induction of this gene during sustained dehydration (Iuchi et al. 2001; Tan et al. 2003), and suggesting some potential redundancy. *AtNCED9* has instead been shown to have an important role in ABA biosynthesis during seed development for induction of dormancy, in accordance with its predominant expression in the embryo and endosperm (Lefebvre et al. 2006).

So far, rapid increases in ABA levels in response to low humidity is a characteristic that has only been observed in angiosperm species. In gymnosperms, although stomata show a strong closure response to ABA, similar to angiosperms (Jackson et al. 1995; Zuccarini et al. 2011; Brodribb and McAdam 2011), increases in ABA levels appear slower, occurring after several hours (McAdam and Brodribb 2014) instead of minutes. Responses of lycophytes, ferns, and gymnosperms to short-term low humidity treatment instead appear to be passively driven by leaf water content, rather than ABA (McAdam and Brodribb 2015). This suggests that mechanisms for rapid ABA-mediated stomatal responses to low humidity may have evolved after angiosperms diverged from gymnosperms, at least 195 million years ago (Morris et al. 2018).

In conclusion, we identified one key *NCED* gene in each eudicot species that is rapidly induced in response to dehydration: *AtNCED3, PsNCED2* and *SlNCED1*, and confirmed the importance of *AtNCED3* in stabilising plant water potential. We found that they all belong to the same major subclade – Core Eudicot NCEDIIB. These results suggest that genes in this subclade share an evolutionarily conserved role in rapid responses to dehydration stress in eudicots. These findings enable future phylogenetically-informed prediction of genes of interest for rapid dehydration responses within this important multigene family in eudicot species.

## Supplementary Information

Below is the link to the electronic supplementary material.

Supplementary file1 (DOCX 2067 KB)

## Data Availability

Sequences for phylogenetics are available at Figshare: 10.6084/m9.figshare.21651833. All other data supporting the findings of this study are available within the paper and its supplementary data.

## Abbreviation

*NCED*: *Nine-cis-Epoxycarotenoid Dioxygenase*
