# Supplementary material for: Dehydration rapidly induces expression of NCED genes from a single subclade in diverse eudicots
Source: Planta. 2025 Jan 28;261(2):46. doi: 10.1007/s00425-025-04626-z (PMC11772543; doi:10.1007/s00425-025-04626-z)
Supplement: Supplementary file 1 — Supplementary file1 (DOCX 2067 KB) [file 425_2025_4626_MOESM1_ESM.docx]

**Dehydration rapidly induces expression of *NCED* genes from a single subclade in diverse eudicots**

Hanh M. Vo, Michael A. Charleston, Timothy J. Brodribb, Frances C. Sussmilch*

School of Natural Sciences, University of Tasmania – Sandy Bay, Tasmania 7005, Australia

*Correspondence: [frances.sussmilch@utas.edu.au](mailto:frances.sussmilch@utas.edu.au); Private Bag 55, Hobart 7001, Tasmania, Australia

Submitted to: *Planta*

**Overview**

**Supplementary Information**

**Suppl. Fig S1** Leaf water potential measurements for the dehydration experiments shown in Fig. 1.

**Suppl. Fig. S2** Expression of *NCED* genes in three studied species *Arabidopsis thaliana* (At), *Pisum sativum* (pea; Ps) and *Solanum lycopersicum* (tomato; Sl) that did not show a significant change in response to decreased water potential from pressurisation treatment (Fig. 1a).

**Suppl. Fig. S3** Expression of NCED genes in three studied species Arabidopsis thaliana (At), Pisum sativum (pea; Ps) and Solanum lycopersicum (tomato; Sl) that did not show a significant change in response to prolonged dehydration (Fig. 1b).

**Suppl. Fig. S4** The importance of the NCED3 gene for stabilising leaf water potential after a rapid drop in humidity.

**Suppl. Fig. S5** Inferred phylogeny of the full *NCED* subfamily in land plants (shown collapsed in Fig. 2).

**Suppl. Table S1** Primer details for qRT-PCR experiments presented in Fig. 1 and Suppl Fig S2 and S3.

**Suppl. Table S2** Gene expression data for Fig. 1a.

**Suppl. Table S3** Gene expression data for Fig. 1b.

**Suppl. Table S4** Details of *NCED* gene sequences used in phylogenetic analysis in Fig. 2 and Suppl Fig. S5.


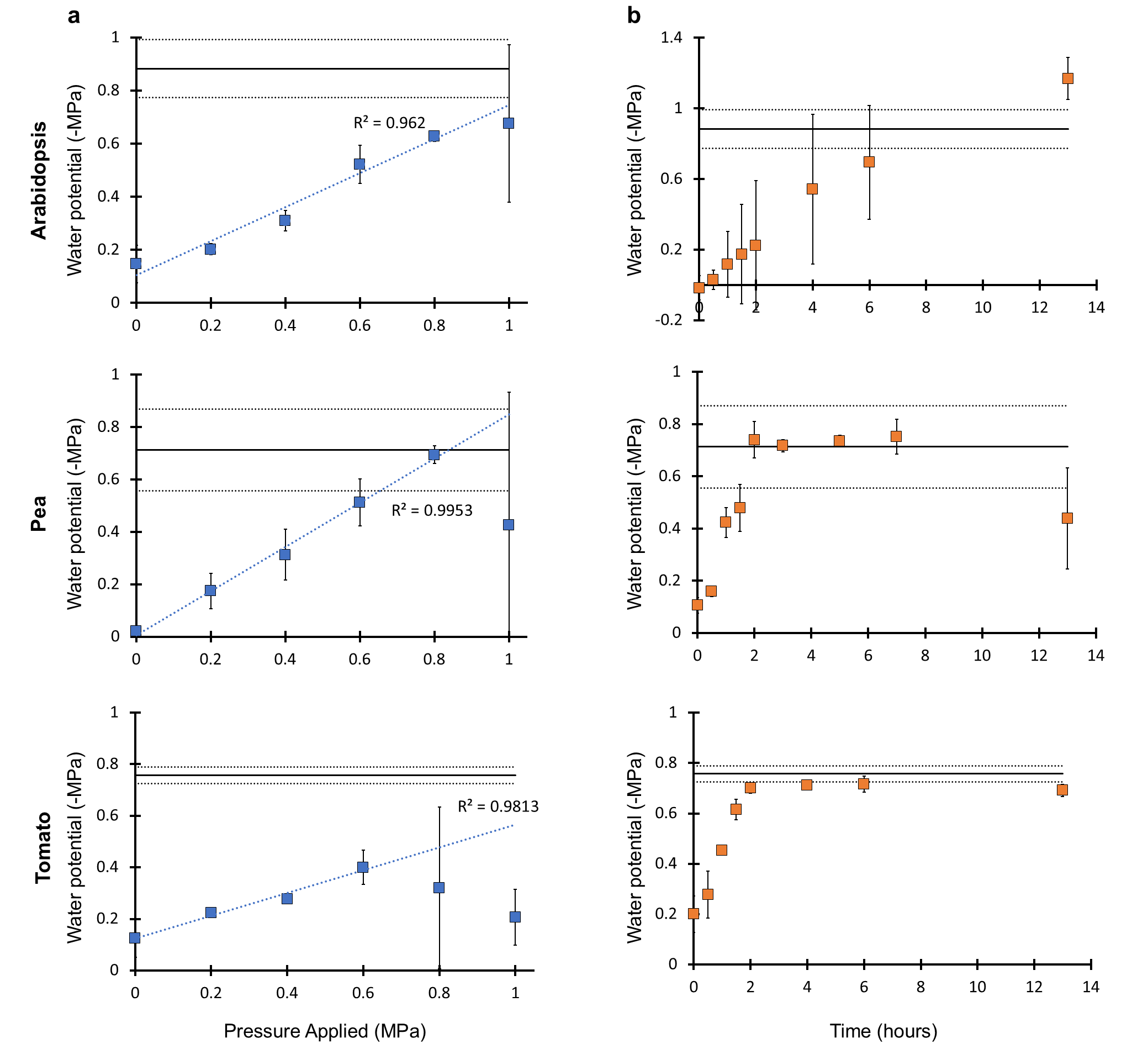


**Suppl. Fig S1 Leaf water potential measurements for the dehydration experiments shown in Fig. 1. a** The linear relationship between the application of external pressure and leaf water potential (experiment shown in **Fig. 1a**). **b** Leaf water potential as plants dried down over 13 hours (experiment shown in **Fig. 1b**) in Arabidopsis*,* pea and tomato. The turgor loss point (TLP) and water potential were measured for each species using a Scholander pressure chamber or psychrometer (Petruzzellis et al., 2019; PSY-1 Stem Psychrometer, ICT International, Armidale, NSW, Autralia). Mean values for water potential (coloured square points) and TLP (solid horizontal line with SD as dotted horizontal line) are shown (*n* = 3 ± SD)*.* In **(a)**, there is a strong linear correlation between external pressure and water potential until high pressure began to cause tissue damage (Rodriguez-Dominguez et al. 2022). Regression was fitted and R^2^ calculated to points before plant cells showed signs of damage. In **(b)**, TLP is reached after two (pea and tomato) or four hours (Arabidopsis) of controlled dehydration.


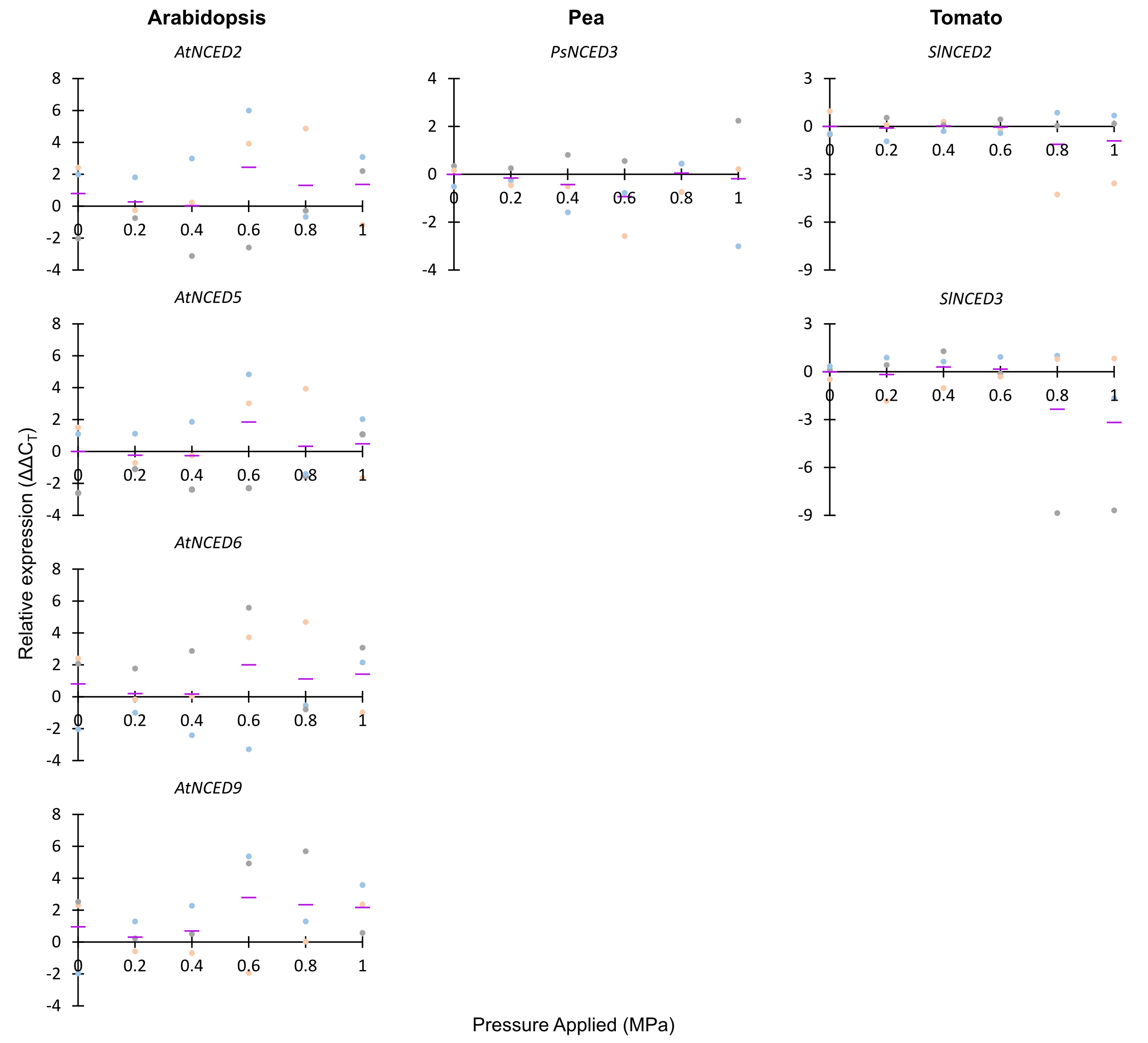


**Suppl. Fig. S2** **Expression of NCED genes in three studied species Arabidopsis thaliana (At), Pisum sativum (pea; Ps) and Solanum lycopersicum (tomato; Sl) that did not show a significant change in response to decreased water potential from pressurisation treatment (Fig. 1a).** Mean expression (purple line) and individual biological replicates (n = 3, coloured points) relative to control values are displayed. One-way ANOVA with Dunnett's multiple comparison test (Dunnett 1964) was performed to compare control (time 0) against each treatment. Full results of statistical tests are given in **Table S2**.

**
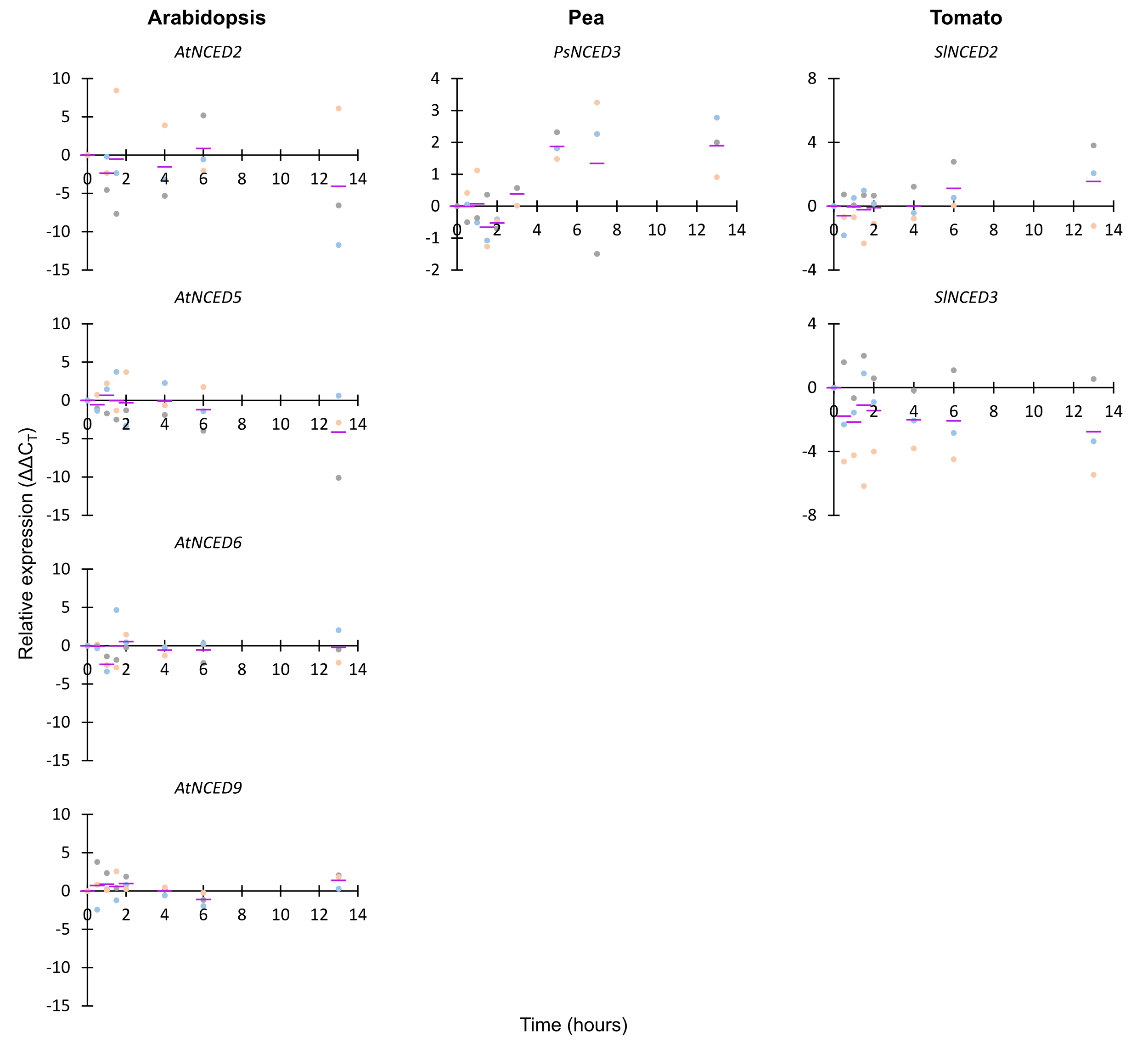
**

**Suppl. Fig. S3** **Expression of NCED genes in three studied species Arabidopsis thaliana (At), Pisum sativum (pea; Ps) and Solanum lycopersicum (tomato; Sl) that did not show a significant change in response to prolonged dehydration** (**Fig. 1b**). Mean expression (purple line) and individual biological replicates (n = 3, coloured points) relative to control values are displayed. One-way ANOVA with Dunnett's multiple comparison test (Dunnett 1964) was performed to compare control (time 0) against each treatment. Full results of statistical tests are given in **Table S3**.


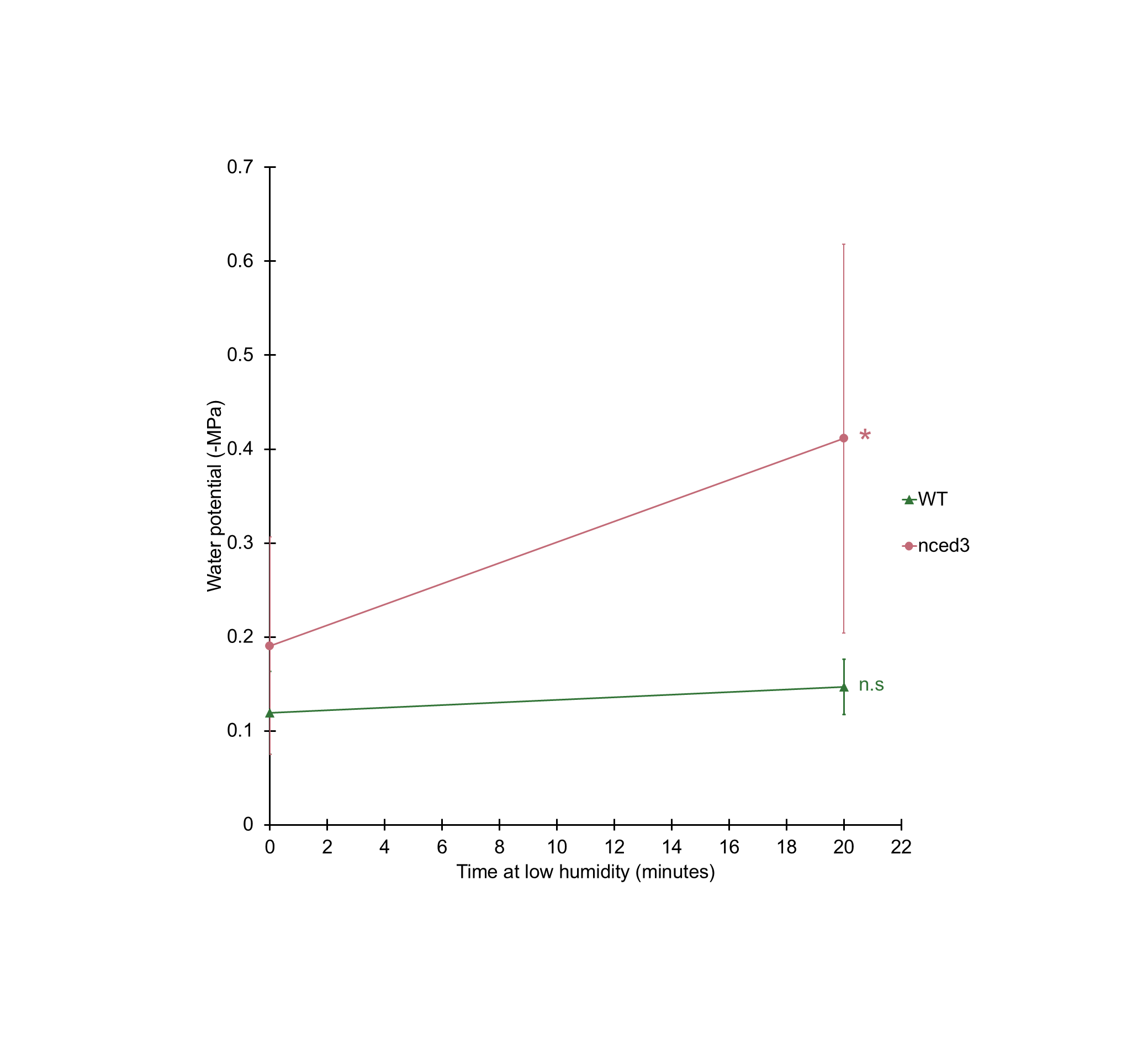


**Suppl. Fig. S4 The importance of the NCED3 gene for stabilising leaf water** **potential after a rapid drop in humidity**. Leaf water potential of 4-week-old nced3-2 mutant (circle) and wild-type (WT, Col-0, triangle) Arabidopsis plants in response to a drop in humidity from 92.17% ± 0.42% to 35.07% ± 6.01% relative humidity at 22.46 ± 0.66 ºC (mean ± SD, n = 8). The asterisk indicates significant differences between WT and nced3 at 20 minutes (P = 0.003), and between 0 and 20 minutes within the nced3 mutant genotype (P = 0.0197) using Dunnett's multiple comparison test (Dunnett 1964). There is no significant difference between 0 and 20 minutes in wild-type plants.


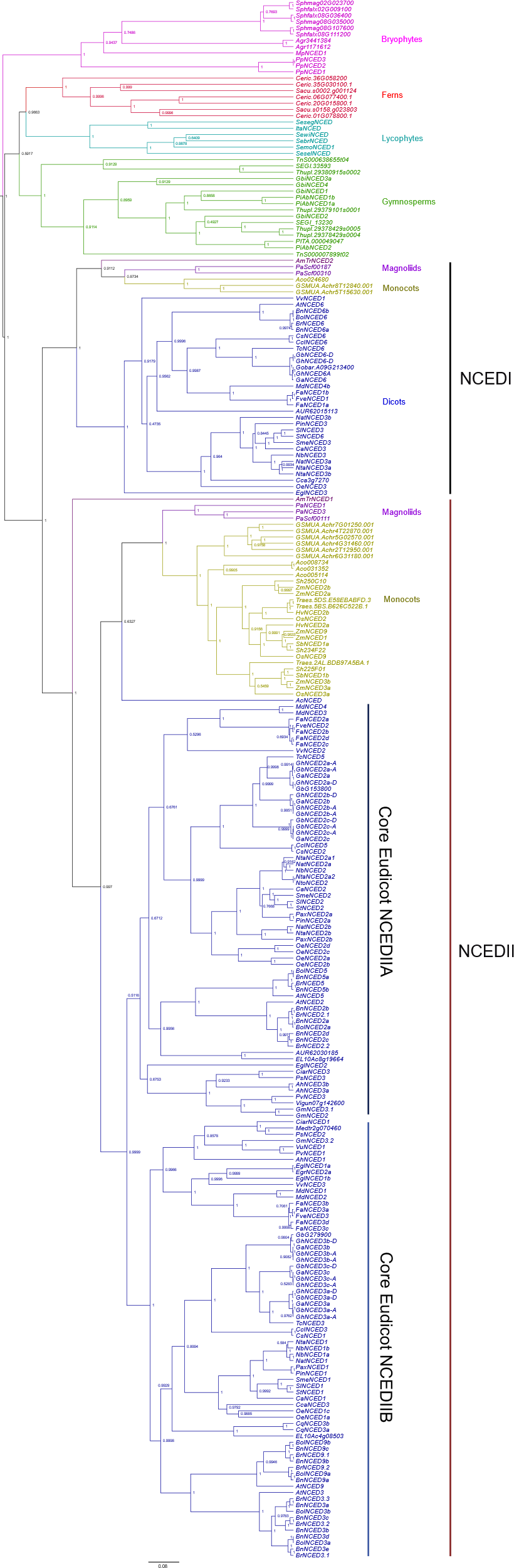


**Suppl. Fig. S5** **Inferred phylogeny of the full *NCED* subfamily in land plants (shown collapsed in** **Fig. 2)**. After alignment of coding sequences using MAFFT translation align (Katoh et al. 2002; Katoh and Standley 2013), the trimmed alignment (available at Figshare: 10.6084/m9.figshare.21651833) was used to construct the consensus tree using Bayesian Evolutionary Analysis by Sampling Trees (BEAST) v1.10.4 (Suchard et al. 2018) using the strict clock model, Yule Process speciation (Gernhard 2008), substitution model GTR + Gamma + invariant sites and estimated base frequencies for 50,000 trees with partition (Lanfear et al. 2012, 2017) and 10% burn-in. Sequence information and additional alignment details are available in **Table S4.** Posterior probability for each node is shown. The scale bar represents number of nucleotide changes per site.

**Suppl. Table S1** **Primer details for qRT-PCR experiments presented in Fig. 1 and Suppl.** **Fig. S2 and S3**. qRT-PCR was performed as previously described (McAdam et al. 2016). Reference genes are indicated with an asterisk (*).

| **Species** | **Gene name** | **Accession number** | **Primer sequences (5' to 3')** | **Tm (°C)** | **Primer source** |
| --- | --- | --- | --- | --- | --- |
| *Pisum sativum* | *PsNCED2* | AB080192/ Psat1g001480 | AGCCGAACCAACTATGATGC | 60 | McAdam, Sussmilch and Brodribb (2016) |
|  |  |  | TTGTCGTAAACAACGGGTGA |  |  |
|  | *PsNCED3* | AB080193/ Psat7g081800 | AAACCGTTGGTCGTTACAGC | 60 | This study |
|  |  |  | TTTTCCGTCTGGTGAGAACC |  |  |
|  | *Helicase (PsHel)** | AY167671 | GCGGGCACTTGGAGATTATC | 59 | Die et al. (2010) |
|  |  |  | ACACTGGTCCCTCCCACACA |  |  |
| *Arabidopsis thaliana* | *AtNCED2* | AT4G18350 | CGGCTGAGCGTGCATTAAT | 60 | Tan et al. (2003) |
|  |  |  | AACACGTGGATCAGCGGTTT |  |  |
|  | *AtNCED3* | AT3G14440 | CGGTGGTTTACGACAAGAACAA | 60 | Barrero et al. (2006) |
|  |  |  | CAGAAGCAATCTGGAGCATCAA |  |  |
|  | *AtNCED5* | AT4G18350 | GACGCCGTAAACTCTCTGCAA | 59 | Tan et al. (2003) |
|  |  |  | TGGAGAAGGGTAGTTTGGGAAA |  |  |
|  | *AtNCED6* | AT3G24220 | CAGAATCGCCCGCTTCCT | 60 | Tan et al. (2003) |
|  |  |  | CGGAGCGAAGTTACCTGATAATTG |  |  |
|  | *AtNCED9* | AT1G78390 | ACGGAGCAAATCCACTTCAC | 60 | Tan et al. (2003) |
|  |  |  | CGACCACATTCACGTTCTTG |  |  |
|  | *MONENSIN SENSITIVITY1 (AtMON1)** | AT2G28390 | AACTCTATGCAGCATTTGATCCACT | 60 | Czechowski et al. (2005) |
|  |  |  | TGATTGCATATCTTTATCGCCATC |  |  |
| *Solanum lycopersicum* | *SlNCED1* | Solyc07g056570 | TCACCGGAAAAATACCCAAA | 60 | McAdam, Sussmilch and Brodribb (2016) |
|  |  |  | GTGAAACGGCAAGCGTAACT |  |  |
|  | *SlNCED2* | Solyc08g016720 | CTCACGCGAACTACAAAACCC | 60 | This study |
|  |  |  | TTTCCGGCGATTTGTACACG |  |  |
|  | *SlNCED3* | Solyc05g053530 | TTGACCCGCCCAAATTAAACC | 60 | This study |
|  |  |  | AAGACCGTGCTGAACAGAGG |  |  |
|  | *SlTIP41-like** | Solyc10g049850 | CATGCCTAGTGGTTGGTTCC | 60 | Zhang et al. (2018a) |
|  |  |  | AGACAAGGCCTGAAATGTGG |  |  |

**Suppl. Table S2** **Gene expression data for** **Fig. 1a**. Relative expression (ΔΔC_T_) for all biological replicates (*n* = 3) are displayed. One-way ANOVA was performed; when ANOVA results showed significant differences between groups, Dunnett's multiple comparison test (Dunnett 1964) was also performed relative to control leaves kept in a bag at 100% humidity.

| **Species** | **Gene** | **Pressure applied (MPa)** | **Replicate** | **Relative expression (ΔΔC_T_)** | **Average expression (ΔΔC_T_)** | **ANOVA *P*-value** | **Dunnet test *P*-value** | **Significance level** |
| --- | --- | --- | --- | --- | --- | --- | --- | --- |
| *Arabidopsis thaliana* | *AtNCED2* | 0 | 1 | -2.023 | 0.794 | 0.917 | **-** |  |
|  |  |  | 2 | 2.418 |  |  |  |  |
|  |  |  | 3 | 1.988 |  |  |  |  |
|  |  | 0.2 | 1 | -0.748 | 0.269 |  | - |  |
|  |  |  | 2 | -0.252 |  |  |  |  |
|  |  |  | 3 | 1.808 |  |  |  |  |
|  |  | 0.4 | 1 | -3.123 | 0.032 |  | - |  |
|  |  |  | 2 | 0.232 |  |  |  |  |
|  |  |  | 3 | 2.988 |  |  |  |  |
|  |  | 0.6 | 1 | -2.593 | 2.439 |  | - |  |
|  |  |  | 2 | 3.918 |  |  |  |  |
|  |  |  | 3 | 5.993 |  |  |  |  |
|  |  | 0.8 | 1 | -0.282 | 1.304 |  | - |  |
|  |  |  | 2 | 4.868 |  |  |  |  |
|  |  |  | 3 | -0.672 |  |  |  |  |
|  |  | 1.0 | 1 | 2.203 | 1.366 |  | - |  |
|  |  |  | 2 | -1.188 |  |  |  |  |
|  |  |  | 3 | 3.083 |  |  |  |  |
| *Arabidopsis thaliana* | *AtNCED3* | 0 | 1 | 0.494 | 0.400 | < 0.001 | 0.884 |  |
|  |  |  | 2 | 0.584 |  |  |  |  |
|  |  |  | 3 | 0.124 |  |  |  |  |
|  |  | 0.2 | 1 | 2.459 | 2.027 |  | 0.015 | * |
|  |  |  | 2 | 2.074 |  |  |  |  |
|  |  |  | 3 | 1.549 |  |  |  |  |
|  |  | 0.4 | 1 | 2.864 | 2.905 |  | < 0.001 | *** |
|  |  |  | 2 | 2.724 |  |  |  |  |
|  |  |  | 3 | 3.129 |  |  |  |  |
|  |  | 0.6 | 1 | 3.484 | 3.375 |  | < 0.001 | *** |
|  |  |  | 2 | 2.559 |  |  |  |  |
|  |  |  | 3 | 4.084 |  |  |  |  |
|  |  | 0.8 | 1 | 4.224 | 3.947 |  | < 0.001 | *** |
|  |  |  | 2 | 2.899 |  |  |  |  |
|  |  |  | 3 | 4.719 |  |  |  |  |
|  |  | 1.0 | 1 | 4.439 | 4.977 |  | < 0.001 | *** |
|  |  |  | 2 | 5.689 |  |  |  |  |
|  |  |  | 3 | 4.804 |  |  |  |  |
| *Arabidopsis thaliana* | *AtNCED5* | 0 | 1 | -2.605 | 0.000 | 0.929 | **-** |  |
|  |  |  | 2 | 1.505 |  |  |  |  |
|  |  |  | 3 | 1.100 |  |  |  |  |
|  |  | 0.2 | 1 | -1.100 | -0.230 |  | - |  |
|  |  |  | 2 | -0.705 |  |  |  |  |
|  |  |  | 3 | 1.115 |  |  |  |  |
|  |  | 0.4 | 1 | -2.385 | -0.255 |  | - |  |
|  |  |  | 2 | -0.245 |  |  |  |  |
|  |  |  | 3 | 1.865 |  |  |  |  |
|  |  | 0.6 | 1 | -2.295 | 1.853 |  | - |  |
|  |  |  | 2 | 3.020 |  |  |  |  |
|  |  |  | 3 | 4.835 |  |  |  |  |
|  |  | 0.8 | 1 | -1.540 | 0.332 |  | - |  |
|  |  |  | 2 | 3.940 |  |  |  |  |
|  |  |  | 3 | -1.405 |  |  |  |  |
|  |  | 1.0 | 1 | 1.075 | 0.487 |  | - |  |
|  |  |  | 2 | -1.645 |  |  |  |  |
|  |  |  | 3 | 2.030 |  |  |  |  |
| *Arabidopsis thaliana* | *AtNCED6* | 0 | 1 | -2.034 | 0.811 | 0.961 | **-** |  |
|  |  |  | 2 | 2.396 |  |  |  |  |
|  |  |  | 3 | 2.071 |  |  |  |  |
|  |  | 0.2 | 1 | -1.004 | 0.201 |  | - |  |
|  |  |  | 2 | -0.164 |  |  |  |  |
|  |  |  | 3 | 1.771 |  |  |  |  |
|  |  | 0.4 | 1 | -2.414 | 0.171 |  | - |  |
|  |  |  | 2 | 0.051 |  |  |  |  |
|  |  |  | 3 | 2.876 |  |  |  |  |
|  |  | 0.6 | 1 | -3.289 | 2.006 |  | - |  |
|  |  |  | 2 | 3.726 |  |  |  |  |
|  |  |  | 3 | 5.581 |  |  |  |  |
|  |  | 0.8 | 1 | -0.544 | 1.120 |  | - |  |
|  |  |  | 2 | 4.691 |  |  |  |  |
|  |  |  | 3 | -0.789 |  |  |  |  |
|  |  | 1.0 | 1 | 2.151 | 1.416 |  | - |  |
|  |  |  | 2 | -0.984 |  |  |  |  |
|  |  |  | 3 | 3.081 |  |  |  |  |
| *Arabidopsis thaliana* | *AtNCED9* | 0 | 1 | 2.306 | 0.960 | 0.673 | **-** |  |
|  |  |  | 2 | 2.536 |  |  |  |  |
|  |  |  | 3 | -1.964 |  |  |  |  |
|  |  | 0.2 | 1 | -0.584 | 0.315 |  | - |  |
|  |  |  | 2 | 0.236 |  |  |  |  |
|  |  |  | 3 | 1.291 |  |  |  |  |
|  |  | 0.4 | 1 | -0.684 | 0.703 |  | - |  |
|  |  |  | 2 | 0.511 |  |  |  |  |
|  |  |  | 3 | 2.281 |  |  |  |  |
|  |  | 0.6 | 1 | -1.934 | 2.791 |  | - |  |
|  |  |  | 2 | 4.931 |  |  |  |  |
|  |  |  | 3 | 5.376 |  |  |  |  |
|  |  | 0.8 | 1 | 0.031 | 2.343 |  | - |  |
|  |  |  | 2 | 5.696 |  |  |  |  |
|  |  |  | 3 | 1.301 |  |  |  |  |
|  |  | 1.0 | 1 | 2.361 | 2.175 |  | - |  |
|  |  |  | 2 | 0.581 |  |  |  |  |
|  |  |  | 3 | 3.581 |  |  |  |  |
| *Solanum lycopersicum* | *SlNCED1* | 0 | 1 | -0.428 | 0.000 | < 0.001 | 1.000 |  |
|  |  |  | 2 | 0.362 |  |  |  |  |
|  |  |  | 3 | 0.067 |  |  |  |  |
|  |  | 0.2 | 1 | 0.737 | 1.057 |  | 0.003 | ** |
|  |  |  | 2 | 1.387 |  |  |  |  |
|  |  |  | 3 | 1.047 |  |  |  |  |
|  |  | 0.4 | 1 | 0.997 | 0.982 |  | 0.005 | ** |
|  |  |  | 2 | 1.147 |  |  |  |  |
|  |  |  | 3 | 0.802 |  |  |  |  |
|  |  | 0.6 | 1 | 2.437 | 1.943 |  | < 0.001 | *** |
|  |  |  | 2 | 1.532 |  |  |  |  |
|  |  |  | 3 | 1.862 |  |  |  |  |
|  |  | 0.8 | 1 | 1.512 | 1.673 |  | < 0.001 | *** |
|  |  |  | 2 | 1.607 |  |  |  |  |
|  |  |  | 3 | 1.902 |  |  |  |  |
|  |  | 1.0 | 1 | 2.402 | 2.150 |  | < 0.001 | *** |
|  |  |  | 2 | 2.117 |  |  |  |  |
|  |  |  | 3 | 1.932 |  |  |  |  |
| *Solanum lycopersicum* | *SlNCED2* | 0 | 1 | 0.952 | 0.000 | 0.901 | **-** |  |
|  |  |  | 2 | -0.503 |  |  |  |  |
|  |  |  | 3 | -0.448 |  |  |  |  |
|  |  | 0.2 | 1 | 0.092 | -0.100 |  | - |  |
|  |  |  | 2 | 0.547 |  |  |  |  |
|  |  |  | 3 | -0.938 |  |  |  |  |
|  |  | 0.4 | 1 | 0.297 | 0.032 |  | - |  |
|  |  |  | 2 | 0.092 |  |  |  |  |
|  |  |  | 3 | -0.293 |  |  |  |  |
|  |  | 0.6 | 1 | -0.158 | -0.047 |  | - |  |
|  |  |  | 2 | 0.442 |  |  |  |  |
|  |  |  | 3 | -0.423 |  |  |  |  |
|  |  | 0.8 | 1 | -4.268 | -1.122 |  | - |  |
|  |  |  | 2 | 0.042 |  |  |  |  |
|  |  |  | 3 | 0.862 |  |  |  |  |
|  |  | 1.0 | 1 | -3.573 | -0.905 |  | - |  |
|  |  |  | 2 | 0.172 |  |  |  |  |
|  |  |  | 3 | 0.687 |  |  |  |  |
| *Solanum lycopersicum* | *SlNCED3* | 0 | 1 | 0.133 | 0.000 | 0.664 | **-** |  |
|  |  |  | 2 | 0.343 |  |  |  |  |
|  |  |  | 3 | -0.477 |  |  |  |  |
|  |  | 0.2 | 1 | 0.428 | -0.170 |  | - |  |
|  |  |  | 2 | 0.888 |  |  |  |  |
|  |  |  | 3 | -1.827 |  |  |  |  |
|  |  | 0.4 | 1 | 1.288 | 0.302 |  | - |  |
|  |  |  | 2 | 0.638 |  |  |  |  |
|  |  |  | 3 | -1.022 |  |  |  |  |
|  |  | 0.6 | 1 | -0.122 | 0.170 |  | - |  |
|  |  |  | 2 | 0.933 |  |  |  |  |
|  |  |  | 3 | -0.302 |  |  |  |  |
|  |  | 0.8 | 1 | -8.857 | -2.348 |  | - |  |
|  |  |  | 2 | 1.008 |  |  |  |  |
|  |  |  | 3 | 0.803 |  |  |  |  |
|  |  | 1.0 | 1 | -8.692 | -3.173 |  | - |  |
|  |  |  | 2 | -1.662 |  |  |  |  |
|  |  |  | 3 | 0.833 |  |  |  |  |
| *Pisum sativum* | *PsNCED2* | 0 | 1 | 0.487 | 0.000 | < 0.001 | 1.000 |  |
|  |  |  | 2 | -0.388 |  |  |  |  |
|  |  |  | 3 | -0.098 |  |  |  |  |
|  |  | 0.2 | 1 | 2.517 | 2.123 |  | 0.125 |  |
|  |  |  | 2 | 2.547 |  |  |  |  |
|  |  |  | 3 | 1.307 |  |  |  |  |
|  |  | 0.4 | 1 | 1.447 | 1.517 |  | 0.372 |  |
|  |  |  | 2 | 1.162 |  |  |  |  |
|  |  |  | 3 | 1.942 |  |  |  |  |
|  |  | 0.6 | 1 | 3.607 | 2.633 |  | 0.044 | * |
|  |  |  | 2 | 1.972 |  |  |  |  |
|  |  |  | 3 | 2.322 |  |  |  |  |
|  |  | 0.8 | 1 | 4.382 | 3.395 |  | 0.009 | ** |
|  |  |  | 2 | 3.787 |  |  |  |  |
|  |  |  | 3 | 2.017 |  |  |  |  |
|  |  | 1.0 | 1 | 9.187 | 7.003 |  | < 0.001 | *** |
|  |  |  | 2 | 7.137 |  |  |  |  |
|  |  |  | 3 | 4.687 |  |  |  |  |
| *Pisum sativum* | *PsNCED3* | 0 | 1 | 0.350 | 0.000 | 0.965 | **-** |  |
|  |  |  | 2 | -0.515 |  |  |  |  |
|  |  |  | 3 | 0.165 |  |  |  |  |
|  |  | 0.2 | 1 | 0.255 | -0.155 |  | - |  |
|  |  |  | 2 | -0.260 |  |  |  |  |
|  |  |  | 3 | -0.460 |  |  |  |  |
|  |  | 0.4 | 1 | 0.810 | -0.428 |  | - |  |
|  |  |  | 2 | -1.590 |  |  |  |  |
|  |  |  | 3 | -0.505 |  |  |  |  |
|  |  | 0.6 | 1 | 0.555 | -0.933 |  | - |  |
|  |  |  | 2 | -0.775 |  |  |  |  |
|  |  |  | 3 | -2.580 |  |  |  |  |
|  |  | 0.8 | 1 | 0.445 | 0.050 |  | - |  |
|  |  |  | 2 | 0.445 |  |  |  |  |
|  |  |  | 3 | -0.740 |  |  |  |  |
|  |  | 1.0 | 1 | 2.240 | -0.185 |  | - |  |
|  |  |  | 2 | -3.010 |  |  |  |  |
|  |  |  | 3 | 0.215 |  |  |  |  |

**Suppl. Table S3** **Gene expression data for Fig. 1b**. Relative expression (ΔΔC_T_) for all biological replicates (*n* = 3) are displayed. One-way ANOVA was performed; when ANOVA results showed significant differences between groups, Dunnett’s multiple comparison test (Dunnett 1964) was also performed relative to the time 0 control (**Fig. 1b**; **Suppl.** **Fig. S3**).

| **Species** | **Gene** | **Time (hours)** | **Replicate** | **Relative expression (ΔΔC_T_)** | **Average expression (ΔΔC_T_)** | **ANOVA *P*-value** | **Dunnet test *P*-value** | **Significance level** |
| --- | --- | --- | --- | --- | --- | --- | --- | --- |
| *Arabidopsis thaliana* | *AtNCED2* | 0 | 1 | 0 | 0 | 0.907 | **-** |  |
|  |  |  | 2 | 0 |  |  |  |  |
|  |  |  | 3 | 0 |  |  |  |  |
|  |  | 1.0 | 1 | -4.545 | -2.357 |  | - |  |
|  |  |  | 2 | -0.195 |  |  |  |  |
|  |  |  | 3 | -2.330 |  |  |  |  |
|  |  | 1.5 | 1 | -7.660 | -0.525 |  | - |  |
|  |  |  | 2 | -2.355 |  |  |  |  |
|  |  |  | 3 | 8.440 |  |  |  |  |
|  |  | 4.0 | 1 | -5.315 | -1.530 |  | - |  |
|  |  |  | 2 | -3.175 |  |  |  |  |
|  |  |  | 3 | 3.900 |  |  |  |  |
|  |  | 6.0 | 1 | 5.190 | 0.873 |  | - |  |
|  |  |  | 2 | -0.555 |  |  |  |  |
|  |  |  | 3 | -2.015 |  |  |  |  |
|  |  | 13.0 | 1 | -6.570 | -4.070 |  | - |  |
|  |  |  | 2 | -11.740 |  |  |  |  |
|  |  |  | 3 | 6.100 |  |  |  |  |
| *Arabidopsis thaliana* | *AtNCED3* | 0 | 1 | 0 | 0 | < 0.001 | **-** |  |
|  |  |  | 2 | 0 |  |  |  |  |
|  |  |  | 3 | 0 |  |  |  |  |
|  |  | 0.5 | 1 | 4.870 | 4.253 |  | < 0.001 | *** |
|  |  |  | 2 | 4.000 |  |  |  |  |
|  |  |  | 3 | 3.890 |  |  |  |  |
|  |  | 1.0 | 1 | 6.025 | 5.132 |  | < 0.001 | *** |
|  |  |  | 2 | 5.365 |  |  |  |  |
|  |  |  | 3 | 4.005 |  |  |  |  |
|  |  | 1.5 | 1 | 5.595 | 5.720 |  | < 0.001 | *** |
|  |  |  | 2 | 4.965 |  |  |  |  |
|  |  |  | 3 | 6.600 |  |  |  |  |
|  |  | 2.0 | 1 | 5.990 | 4.360 |  | < 0.001 | *** |
|  |  |  | 2 | 3.120 |  |  |  |  |
|  |  |  | 3 | 3.970 |  |  |  |  |
|  |  | 4.0 | 1 | 4.605 | 3.883 |  | < 0.001 | *** |
|  |  |  | 2 | 2.795 |  |  |  |  |
|  |  |  | 3 | 4.250 |  |  |  |  |
|  |  | 6.0 | 1 | 5.525 | 5.165 |  | < 0.001 | *** |
|  |  |  | 2 | 4.910 |  |  |  |  |
|  |  |  | 3 | 5.060 |  |  |  |  |
|  |  | 13.0 | 1 | 3.580 | 3.657 |  | < 0.001 | *** |
|  |  |  | 2 | 3.640 |  |  |  |  |
|  |  |  | 3 | 3.750 |  |  |  |  |
| *Arabidopsis thaliana* | *AtNCED5* | 0 | 1 | 0 | 0 | 0.651 | **-** |  |
|  |  |  | 2 | 0 |  |  |  |  |
|  |  |  | 3 | 0 |  |  |  |  |
|  |  | 0.5 | 1 | 0.740 | -0.550 |  | - |  |
|  |  |  | 2 | -1.050 |  |  |  |  |
|  |  |  | 3 | -1.340 |  |  |  |  |
|  |  | 1.0 | 1 | 2.245 | 0.672 |  | - |  |
|  |  |  | 2 | -1.695 |  |  |  |  |
|  |  |  | 3 | 1.465 |  |  |  |  |
|  |  | 1.5 | 1 | -1.315 | -0.022 |  | - |  |
|  |  |  | 2 | -2.495 |  |  |  |  |
|  |  |  | 3 | 3.745 |  |  |  |  |
|  |  | 2.0 | 1 | 3.710 | -0.290 |  | - |  |
|  |  |  | 2 | -1.285 |  |  |  |  |
|  |  |  | 3 | -3.295 |  |  |  |  |
|  |  | 4.0 | 1 | -0.625 | -0.075 |  | - |  |
|  |  |  | 2 | -1.890 |  |  |  |  |
|  |  |  | 3 | 2.290 |  |  |  |  |
|  |  | 6.0 | 1 | 1.755 | -1.200 |  | - |  |
|  |  |  | 2 | -3.955 |  |  |  |  |
|  |  |  | 3 | -1.400 |  |  |  |  |
|  |  | 13.0 | 1 | -2.890 | -4.120 |  | - |  |
|  |  |  | 2 | -10.100 |  |  |  |  |
|  |  |  | 3 | 0.630 |  |  |  |  |
| *Arabidopsis thaliana* | *AtNCED6* | 0 | 1 | 0 | 0 | 0.641 | **-** |  |
|  |  |  | 2 | 0 |  |  |  |  |
|  |  |  | 3 | 0 |  |  |  |  |
|  |  | 0.5 | 1 | 0.165 | -0.077 |  | - |  |
|  |  |  | 2 | -0.060 |  |  |  |  |
|  |  |  | 3 | -0.335 |  |  |  |  |
|  |  | 1.0 | 1 | -2.525 | -2.428 |  | - |  |
|  |  |  | 2 | -1.395 |  |  |  |  |
|  |  |  | 3 | -3.365 |  |  |  |  |
|  |  | 1.5 | 1 | -2.845 | -0.008 |  | - |  |
|  |  |  | 2 | -1.845 |  |  |  |  |
|  |  |  | 3 | 4.665 |  |  |  |  |
|  |  | 2.0 | 1 | 1.450 | 0.548 |  | - |  |
|  |  |  | 2 | -0.235 |  |  |  |  |
|  |  |  | 3 | 0.430 |  |  |  |  |
|  |  | 4.0 | 1 | -1.290 | -0.565 |  | - |  |
|  |  |  | 2 | -0.205 |  |  |  |  |
|  |  |  | 3 | -0.200 |  |  |  |  |
|  |  | 6.0 | 1 | 0.250 | -0.557 |  | - |  |
|  |  |  | 2 | -2.245 |  |  |  |  |
|  |  |  | 3 | 0.325 |  |  |  |  |
|  |  | 13.0 | 1 | -2.205 | -0.223 |  | - |  |
|  |  |  | 2 | -0.500 |  |  |  |  |
|  |  |  | 3 | 2.035 |  |  |  |  |
| *Arabidopsis thaliana* | *AtNCED9* | 0 | 1 | 0 | 0 | 0.570 | **-** |  |
|  |  |  | 2 | 0 |  |  |  |  |
|  |  |  | 3 | 0 |  |  |  |  |
|  |  | 0.5 | 1 | 3.800 | 0.735 |  | - |  |
|  |  |  | 2 | -2.425 |  |  |  |  |
|  |  |  | 3 | 0.830 |  |  |  |  |
|  |  | 1.0 | 1 | 2.345 | 0.897 |  | - |  |
|  |  |  | 2 | 0.230 |  |  |  |  |
|  |  |  | 3 | 0.115 |  |  |  |  |
|  |  | 1.5 | 1 | 0.410 | 0.597 |  | - |  |
|  |  |  | 2 | -1.200 |  |  |  |  |
|  |  |  | 3 | 2.580 |  |  |  |  |
|  |  | 2.0 | 1 | 1.885 | 0.987 |  | - |  |
|  |  |  | 2 | 0.810 |  |  |  |  |
|  |  |  | 3 | 0.265 |  |  |  |  |
|  |  | 4.0 | 1 | 0.155 | 0.023 |  | - |  |
|  |  |  | 2 | -0.580 |  |  |  |  |
|  |  |  | 3 | 0.495 |  |  |  |  |
|  |  | 6.0 | 1 | -1.160 | -1.103 |  | - |  |
|  |  |  | 2 | -1.950 |  |  |  |  |
|  |  |  | 3 | -0.200 |  |  |  |  |
|  |  | 13.0 | 1 | 2.060 | 1.403 |  | - |  |
|  |  |  | 2 | 0.310 |  |  |  |  |
|  |  |  | 3 | 1.840 |  |  |  |  |
| *Solanum lycopersicum* | *SlNCED1* | 0 | 1 | 0 | 0 | < 0.001 | **-** |  |
|  |  |  | 2 | 0 |  |  |  |  |
|  |  |  | 3 | 0 |  |  |  |  |
|  |  | 0.5 | 1 | 3.745 | 2.638 |  | 0.022 | * |
|  |  |  | 2 | 3.160 |  |  |  |  |
|  |  |  | 3 | 1.010 |  |  |  |  |
|  |  | 1.0 | 1 | 1.615 | 1.722 |  | 0.193 |  |
|  |  |  | 2 | 3.280 |  |  |  |  |
|  |  |  | 3 | 0.270 |  |  |  |  |
|  |  | 1.5 | 1 | 1.600 | 2.068 |  | 0.089 |  |
|  |  |  | 2 | 1.210 |  |  |  |  |
|  |  |  | 3 | 3.395 |  |  |  |  |
|  |  | 2.0 | 1 | 3.160 | 2.830 |  | 0.013 | * |
|  |  |  | 2 | 3.230 |  |  |  |  |
|  |  |  | 3 | 2.100 |  |  |  |  |
|  |  | 4.0 | 1 | 5.025 | 5.058 |  | < 0.001 | *** |
|  |  |  | 2 | 5.845 |  |  |  |  |
|  |  |  | 3 | 4.305 |  |  |  |  |
|  |  | 6.0 | 1 | 4.890 | 4.455 |  | < 0.001 | *** |
|  |  |  | 2 | 4.040 |  |  |  |  |
|  |  |  | 3 | 4.435 |  |  |  |  |
|  |  | 13.0 | 1 | 5.035 | 4.905 |  | < 0.001 | *** |
|  |  |  | 2 | 4.085 |  |  |  |  |
|  |  |  | 3 | 5.595 |  |  |  |  |
| *Solanum lycopersicum* | *SlNCED2* | 0 | 1 | 0 | 0 | 0.608 | **-** |  |
|  |  |  | 2 | 0 |  |  |  |  |
|  |  |  | 3 | 0 |  |  |  |  |
|  |  | 0.5 | 1 | 0.730 | -0.593 |  | - |  |
|  |  |  | 2 | -0.680 |  |  |  |  |
|  |  |  | 3 | -1.830 |  |  |  |  |
|  |  | 1.0 | 1 | 0.060 | -0.038 |  | - |  |
|  |  |  | 2 | -0.695 |  |  |  |  |
|  |  |  | 3 | 0.520 |  |  |  |  |
|  |  | 1.5 | 1 | 0.690 | -0.218 |  | - |  |
|  |  |  | 2 | -2.330 |  |  |  |  |
|  |  |  | 3 | 0.985 |  |  |  |  |
|  |  | 2.0 | 1 | 0.660 | -0.093 |  | - |  |
|  |  |  | 2 | -1.095 |  |  |  |  |
|  |  |  | 3 | 0.155 |  |  |  |  |
|  |  | 4.0 | 1 | 1.225 | 0.010 |  | - |  |
|  |  |  | 2 | -0.775 |  |  |  |  |
|  |  |  | 3 | -0.420 |  |  |  |  |
|  |  | 6.0 | 1 | 2.785 | 1.120 |  | - |  |
|  |  |  | 2 | 0.045 |  |  |  |  |
|  |  |  | 3 | 0.530 |  |  |  |  |
|  |  | 13.0 | 1 | 3.810 | 1.547 |  | - |  |
|  |  |  | 2 | -1.235 |  |  |  |  |
|  |  |  | 3 | 2.065 |  |  |  |  |
| *Solanum lycopersicum* | *SlNCED3* | 0 | 1 | 0 | 0 | 0.952 | **-** |  |
|  |  |  | 2 | 0 |  |  |  |  |
|  |  |  | 3 | 0 |  |  |  |  |
|  |  | 0.5 | 1 | 1.600 | -1.780 |  | - |  |
|  |  |  | 2 | -4.630 |  |  |  |  |
|  |  |  | 3 | -2.310 |  |  |  |  |
|  |  | 1.0 | 1 | -0.655 | -2.148 |  | - |  |
|  |  |  | 2 | -4.230 |  |  |  |  |
|  |  |  | 3 | -1.560 |  |  |  |  |
|  |  | 1.5 | 1 | 2.005 | -1.090 |  | - |  |
|  |  |  | 2 | -6.170 |  |  |  |  |
|  |  |  | 3 | 0.895 |  |  |  |  |
|  |  | 2.0 | 1 | 0.590 | -1.437 |  | - |  |
|  |  |  | 2 | -4.005 |  |  |  |  |
|  |  |  | 3 | -0.895 |  |  |  |  |
|  |  | 4.0 | 1 | -0.165 | -2.010 |  | - |  |
|  |  |  | 2 | -3.805 |  |  |  |  |
|  |  |  | 3 | -2.060 |  |  |  |  |
|  |  | 6.0 | 1 | 1.095 | -2.075 |  | - |  |
|  |  |  | 2 | -4.485 |  |  |  |  |
|  |  |  | 3 | -2.835 |  |  |  |  |
|  |  | 13.0 | 1 | 0.550 | -2.755 |  | - |  |
|  |  |  | 2 | -5.460 |  |  |  |  |
|  |  |  | 3 | -3.355 |  |  |  |  |
| *Pisum sativum* | *PsNCED2* | 0 | 1 | 0 | 0 | < 0.001 | **-** |  |
|  |  |  | 2 | 0 |  |  |  |  |
|  |  |  | 3 | 0 |  |  |  |  |
|  |  | 0.5 | 1 | 4.415 | 3.668 |  | 0.015 | * |
|  |  |  | 2 | 5.295 |  |  |  |  |
|  |  |  | 3 | 1.295 |  |  |  |  |
|  |  | 1.0 | 1 | 5.640 | 5.423 |  | < 0.001 | *** |
|  |  |  | 2 | 6.020 |  |  |  |  |
|  |  |  | 3 | 4.610 |  |  |  |  |
|  |  | 1.5 | 1 | 8.020 | 7.032 |  | < 0.001 | *** |
|  |  |  | 2 | 6.915 |  |  |  |  |
|  |  |  | 3 | 6.160 |  |  |  |  |
|  |  | 2.0 | 1 | 7.630 | 8.140 |  | < 0.001 | *** |
|  |  |  | 2 | 7.190 |  |  |  |  |
|  |  |  | 3 | 9.600 |  |  |  |  |
|  |  | 3.0 | 1 | 11.515 | 10.082 |  | < 0.001 | *** |
|  |  |  | 2 | 7.465 |  |  |  |  |
|  |  |  | 3 | 11.265 |  |  |  |  |
|  |  | 5.0 | 1 | 7.315 | 7.422 |  | < 0.001 | *** |
|  |  |  | 2 | 8.015 |  |  |  |  |
|  |  |  | 3 | 6.935 |  |  |  |  |
|  |  | 7.0 | 1 | 9.510 | 8.303 |  | < 0.001 | *** |
|  |  |  | 2 | 8.470 |  |  |  |  |
|  |  |  | 3 | 6.930 |  |  |  |  |
|  |  | 13.0 | 1 | 6.675 | 6.847 |  | < 0.001 | *** |
|  |  |  | 2 | 7.060 |  |  |  |  |
|  |  |  | 3 | 6.805 |  |  |  |  |
| *Pisum sativum* | *PsNCED3* | 0 | 1 | 0 | 0 | 0.034 | **-** |  |
|  |  |  | 2 | 0 |  |  |  |  |
|  |  |  | 3 | 0 |  |  |  |  |
|  |  | 0.5 | 1 | 0.055 | -0.010 |  | 1.000 |  |
|  |  |  | 2 | 0.415 |  |  |  |  |
|  |  |  | 3 | -0.500 |  |  |  |  |
|  |  | 1.0 | 1 | -0.515 | 0.078 |  | 1.000 |  |
|  |  |  | 2 | 1.120 |  |  |  |  |
|  |  |  | 3 | -0.370 |  |  |  |  |
|  |  | 1.5 | 1 | -1.075 | -0.660 |  | 0.957 |  |
|  |  |  | 2 | -1.265 |  |  |  |  |
|  |  |  | 3 | 0.360 |  |  |  |  |
|  |  | 2.0 | 1 | -0.410 | -0.527 |  | 0.987 |  |
|  |  |  | 2 | -0.465 |  |  |  |  |
|  |  |  | 3 | -0.705 |  |  |  |  |
|  |  | 3.0 | 1 | 0.570 | 0.385 |  | 0.998 |  |
|  |  |  | 2 | 0.015 |  |  |  |  |
|  |  |  | 3 | 0.570 |  |  |  |  |
|  |  | 5.0 | 1 | 1.810 | 1.870 |  | 0.180 |  |
|  |  |  | 2 | 1.480 |  |  |  |  |
|  |  |  | 3 | 2.320 |  |  |  |  |
|  |  | 7.0 | 1 | 2.265 | 1.340 |  | 0.484 |  |
|  |  |  | 2 | 3.250 |  |  |  |  |
|  |  |  | 3 | -1.495 |  |  |  |  |
|  |  | 13.0 | 1 | 2.775 | 1.895 |  | 0.171 |  |
|  |  |  | 2 | 0.910 |  |  |  |  |
|  |  |  | 3 | 2.000 |  |  |  |  |

**Suppl. Table S4** **Details of *NCED* gene sequences used in phylogenetic analysis in Fig. 2** **and** **Suppl.** **Fig. S5**. Sequences were obtained from: Phytozome v13 (https://phytozome-next.jgi.doe.gov/ ; Goodstein et al., 2012), GenBank (Clark et al. 2016; Sayers et al. 2022), ConGenIE (Nystedt et al. 2013; Sundell et al. 2015), Sol Genomics Network (Fernandez-Pozo et al. 2015), FernBase (Li et al. 2018), TreeGenesdb (Wegrzyn et al. 2008), OneKP (Leebens-Mack et al. 2019) and CoGe (Lyons and Freeling 2008; Lyons et al. 2008), unless specified otherwise. Coding sequences (CDS) were obtained using BLASTN with *AtNCED3* CDS as query and confirmed to be *NCED* genes through reciprocal BLAST against Arabidopsis and preliminary phylogenetic analysis. Sequences were aligned using MAFFT translation align (Katoh et al. 2002; Katoh and Standley 2013) with gap opening penalty of 3 and an offset value of 0.123. Partial sequences and sites with coverage of less than 97% of gene sequences were eliminated, leaving 1842 positions for use in phylogenetic analyses (trimmed alignment available at Figshare: 10.6084/m9.figshare.21651833).

| **Plant lineage** | **Species** | **Gene name** | **Gene accession/ID** | **Source** | **Version** | **Reference** |
| --- | --- | --- | --- | --- | --- | --- |
| Bryophytes | *Anthoceros agrestis* | *-* | Agr1171612 | [UZH - Hornworts - Anthoceros genomes (https://www.hornworts.uzh.ch/en/hornwort-genomes.html)](https://www.hornworts.uzh.ch/en/hornwort-genomes.html) | *A. agrestis* [bonn] | Li et al. (2020) |
|  |  | *-* | Agr3441384 |  |  |  |
|  | *Marchantia polymorpha* | *MpNCED1* | Mapoly0015s0066 | Phytozome v13 | v3.1 | Bowman et al. (2017) |
|  | *Physcomitrium* (prev. *Physcomitrella*) *patens* | *PpNCED1* | Pp3c16.17210V3.3 | Phytozome v13 | v3.3 | Lang et al. (2018) |
|  |  | *PpNCED2* | Pp3c25.4816V3.3 |  |  |  |
|  |  | *PpNCED3* | Pp3c25.4810V3.3 |  |  |  |
|  | *Sphagnum fallax* | - | Sphfalx02G009100 | Phytozome v13 | v1.1 | DOE-JGI, http://phytozome.jgi.doe.gov/ |
|  |  | *-* | Sphfalx08G036400 |  |  |  |
|  |  | *-* | Sphfalx08G111200 |  |  |  |
|  | *Sphagnum magellanicum* | *-* | Sphmag02G023700 | Phytozome v13 | v1.1 | DOE-JGI, http://phytozome.jgi.doe.gov/ |
|  |  | *-* | Sphmag08G035000 |  |  |  |
|  |  | *-* | Sphmag08G107600 |  |  |  |
| Lycophytes | *Isoetes taiwanensis* | *ItaNCED* | Itaiw.v1.scaffold.106 1329864 | CoGe | *Isoetes taiwanensis* (vv.submission, id61511) | Wickell et al. (2021) |
|  | *Selaginella bryopteris* | *SebrNCED* | GEMU01096721.1 | OneKP transcriptome sequence | - | Leebens-Mack et al. (2019) |
|  | *Selaginella moellendorffii* | *SemoNCED1* | Selaginella.moellendorffii.233638 | Phytozome v13 | v1.0 | Banks et al. (2011) |
|  | *Selaginella selaginoides* | *SesegNCED* | KUXM.scaffold.2007655 | OneKP transcriptome sequence | - | Leebens-Mack et al. (2019) |
|  | *Selaginella sellowii* | *SeselNCED* | GIMF01010786.1 | OneKP transcriptome sequence | - | Leebens-Mack et al. (2019) |
|  | *Selaginella willdenowii* | *SewiNCED* | KJYC.scaffold.2007130 | OneKP transcriptome sequence | - | Leebens-Mack et al. (2019) |
| Gymnosperms | *Gingko biloba* | *GbiNCED1* | Gbichr12.1566 | GenBank, Bioproject: PRJCA001755 | HiC.CDS | Liu et al. (2021) |
|  |  | *GbiNCED2* | Gbichr10.706 |  |  |  |
|  |  | *GbiNCED3a* | Gbichr1.2985 |  |  |  |
|  |  | *GbiNCED4* | Gbchr1.2480 |  |  |  |
|  | *Gnetum montanum* | *-* | TnS000007899t02 | Genbank, Bioproject PRJNA339497 | PRJNA339497 | Wan et al. (2018) |
|  |  | *-* | TnS000638655t04 |  |  |  |
|  | *Picea abies* | *PiAbNCED1a* | MA.10428505g0020 | ConGenIE | v1 | Nystedt et al. (2013); Sundell et al. (2015) |
|  |  | *PiAbNCED1b* | MA.10428505g0010 |  |  |  |
|  |  | *PiAbNCED2* | MA.10434448g0010 |  |  |  |
|  | *Pinus taeda* |  | PITA.000049047 | ConGenIE | v2.01 | Nystedt et al. (2013); Sundell et al. (2015) |
|  | *Sequoiadendron giganteum* | *-* | SEGI.13230 | TreeGenesdb | v2.0 | Falk et al. (2018); Wegrzyn et al. (2019) |
|  |  | *-* | SEGI.33593 |  |  |  |
|  | *Thuja plicata* | *-* | Thupl.29378429s0004 | Phytozome v13 | v3.1 | DOE-JGI, http://phytozome-next.jgi.doe.gov/ |
|  |  | *-* | Thupl.29378429s0005 |  |  |  |
|  |  | *-* | Thupl.29379101s0001 |  |  |  |
|  |  | *-* | Thupl.29380915s0002 |  |  |  |
| Ferns | *Ceratopteris richardii* | *-* | Ceric.01G078800.1 | Phytozome v13 | v2.1 | Marchant et al. (2022) |
|  |  | *-* | Ceric.06G077400.1 |  |  |  |
|  |  | *-* | Ceric.20G015800.1 |  |  |  |
|  |  | *-* | Ceric.35G030100.1 |  |  |  |
|  |  | *-* | Ceric.36G058200.1 |  |  |  |
|  | *Salvinia cucullata* | *-* | Sacu.s0002.g001124 | FernBase | v1.1 | Li et al. (2018) |
|  |  | *-* | Sacu.s0158.g023803 |  |  |  |
| “Basal” angiosperm | *Amborella trichopoda* | *AmtrNCED1* | evm.27.model.AmTr.v1.0.scaffold00092.158 | Phytozome v13 | v1.0 | Albert et al. (2013) |
|  |  | *AmtrNCED2* | evm.27.model.AmTr.v1.0.scaffold00039.158 |  |  |  |
| Monocots | *Ananas comosus* | *-* | Aco024680 | Phytozome v13 | v3 | Ming et al. (2015) |
|  |  | *-* | Aco031352 |  |  |  |
|  |  | *-* | Aco005114 |  |  |  |
|  |  | *-* | Aco008734 |  |  |  |
|  | *Hordeum vulgare* | *HvNCED2a* | HORVU5Hr1G092850 | Phytozome v13 | r1 | Beier et al. (2017) |
|  |  | *HvNCED2b* | HORVU5Hr1G008050 |  |  |  |
|  | *Musa acuminata* | *-* | GSMUA.Achr2T12950 | Phytozome v13 | v1 | D’Hont et al. (2012) |
|  |  | *-* | GSMUA.Achr4G31460 |  |  |  |
|  |  | *-* | GSMUA.Achr4T22870 |  |  |  |
|  |  | *-* | GSMUA.Achr5G02570 |  |  |  |
|  |  | *-* | GSMUA.Achr5T15630 |  |  |  |
|  |  | *-* | GSMUA.Achr6G31180 |  |  |  |
|  |  | *-* | GSMUA.Achr7G01250 |  |  |  |
|  |  | *-* | GSMUA.Achr8T12840 |  |  |  |
|  | *Oryza sativa* | *OsNCED2* | Os12g42280 | Phytozome v13 | v7.0 | Ouyang et al. (2007) |
|  |  | *OsNCED3a* | Os07g05940 |  |  |  |
|  |  | *OsNCED9* | Os03g44380 |  |  |  |
|  | *Saccharum hybrid* | *-* | Sh225F01 | Sugarcane Genome Hub | Sugarcane BAC | Garsmeur et al. (2018) |
|  |  | *-* | Sh234F22 |  |  |  |
|  |  | *-* | Sh250C10 |  |  |  |
|  | *Sorghum bicolor* | *SbNCED1a* | Sobic.001G155300 | Phytozome v13 | v3.1.1 | McCormick et al. (2018) |
|  |  | *SbNCED1b* | Sobic.002G037400 |  |  |  |
|  | *Triticum aestivum* | *-* | Traes.2AL.BDB97A5BA | Phytozome v13 | v2.2 | Mayer et al. (2014) |
|  |  | *-* | Traes.5BS.B626C522B |  |  |  |
|  |  | *-* | Traes.5DS.E58EBABFD |  |  |  |
|  | *Zea mays* | *ZmNCED1* | Zm00001d033222 | Phytozome v13 | RefGen.V4 | Schnable et al. (2009) |
|  |  | *ZmNCED2a* | Zm00001d041319 |  |  |  |
|  |  | *ZmNCED2b* | Zm00001d031086 |  |  |  |
|  |  | *ZmNCED3a* | Zm00001d018819 |  |  |  |
|  |  | *ZmNCED3b* | Zm00001d007876 |  |  |  |
|  |  | *ZmNCED9* | Zm00001d013689 |  |  |  |
| Magnoliids | *Persea americana* (Hass) | *PaNCED1* | PaScf00270 | CoGe | v2.0 | Rendón-Anaya et al. (2019) |
|  |  | *PaNCED3* | PaScf00010 |  |  |  |
|  |  | *-* | PaScf00111 |  |  |  |
|  |  | *-* | PaScf00187 |  |  |  |
|  |  | *-* | PaScf00310 |  |  |  |
| Eudicots | *Aquilegia coerulea* | *AcNCED* | Aqcoe4G000100 | Phytozome v13 | v3.1 | Filiault et al. (2018) |
|  | *Arabidopsis thaliana* | *AtNCED2* | AT4G18350 | Phytozome v13 | Araport11 | Cheng et al. (2017) |
|  |  | *AtNCED3* | AT3G14440 |  |  |  |
|  |  | *AtNCED5* | AT1G30100 |  |  |  |
|  |  | *AtNCED6* | AT3G24220 |  |  |  |
|  |  | *AtNCED9* | AT1G78390 |  |  |  |
|  | *Arachis hypogaea* | *AhNCED1* | AJ574819 | Phytozome v13 | v1.0 | Bertioli et al. (2019) |
|  |  | *AhNCED3a* | arahy.Tifrunner.gnm1.ann1.8YR14Z.1 |  |  |  |
|  |  | *AhNCED3b* | arahy.Tifrunner.gnm1.ann1.2M7K4U.1 |  |  |  |
|  | *Beta vulgaris* | *-* | EL10Ac4g08503.1 | Phytozome v13 | EL10.1.0 | McGrath et al. (2022) |
|  |  | *-* | EL10Ac8g19664.1 |  |  |  |
|  | *Brassica napus* | *BnNCED2a* | Bna.C01p010040.1 | Zenodo, supplement to PRJNA587046 | Express617.v1 | Lee et al. (2020) |
|  |  | *BnNCED2b* | Bna.A01p009180.1 |  |  |  |
|  |  | *BnNCED2c* | Bna.C07p028450.1 |  |  |  |
|  |  | *BnNCED2d* | Bna.A03p044410.1 |  |  |  |
|  |  | *BnNCED3a* | Bna.A03p033930.1 |  |  |  |
|  |  | *BnNCED3b* | Bna.C01p032730.1 |  |  |  |
|  |  | *BnNCED3c* | Bna.A01p033240.1 |  |  |  |
|  |  | *BnNCED3d* | Bna.C05p036520.1 |  |  |  |
|  |  | *BnNCED3e* | Bna.A05p028340.2 |  |  |  |
|  |  | *BnNCED5a* | Bna.C05p024270.1 |  |  |  |
|  |  | *BnNCED5b* | Bna.A09p028090.1 |  |  |  |
|  |  | *BnNCED6a* | Bna.A07p006050.1 |  |  |  |
|  |  | *BnNCED6b* | Bna.C07p001970.1 |  |  |  |
|  |  | *BnNCED9a* | Bna.C02p044650.1 |  |  |  |
|  |  | *BnNCED9b* | Bna.A07p035950.1 |  |  |  |
|  |  | *BnNCED9c* | Bna.C06p040050.1 |  |  |  |
|  | *Brassica oleracea* | *BolNCED2a* | Bol009433 | Phytozome v13 | v1.0 | Liu et al. (2014) |
|  |  | *BolNCED3a* | Bol005093 |  |  |  |
|  |  | *BolNCED3b* | Bol035582 |  |  |  |
|  |  | *BolNCED5* | Bol022516 |  |  |  |
|  |  | *BolNCED6* | Bol007451 |  |  |  |
|  |  | *BolNCED9a* | Bol018961 |  |  |  |
|  |  | *BolNCED9b* | Bol027485 |  |  |  |
|  | *Brassica rapa* | *BrNCED2.1* | Brara.A00958.1 | Phytozome v13 | B.rapaFPsc v1.3 | Zhang et al. (2018b) |
|  |  | *BrNCED2.2* | Brara.K00450.1 |  |  |  |
|  |  | *BrNCED3.1* | Brara.E02677.1 |  |  |  |
|  |  | *BrNCED3.2* | Brara.A03189.1 |  |  |  |
|  |  | *BrNCED3.3* | Brara.C03527.1 |  |  |  |
|  |  | *BrNCED5* | Brara.I02844.1 |  |  |  |
|  |  | *BrNCED6* | Brara.G00531.1 |  |  |  |
|  |  | *BrNCED9.1* | Brara.G03580.1 |  |  |  |
|  |  | *BrNCED9.2* | Brara.B02322.1 |  |  |  |
|  | *Capsicum annuum* | *CaNCED1* | Capang07g002066 | Sol Genomics Network | CM334 Genome CDS (release 1.55) | Kim et al. (2014) |
|  |  | *CaNCED2* | Capang00g002048 |  |  |  |
|  |  | *CaNCED3* | Capang05g001826 |  |  |  |
|  | *Chenopodium quinoa* | *-* | AUR62015113 | Phytozome v13 | v1.0 | Jarvis et al. (2017) |
|  |  | *-* | AUR62030185 |  |  |  |
|  |  | *CqNCED3a* | AUR62043087 |  |  |  |
|  |  | *CqNCED3b* | AUR62037951 |  |  |  |
|  | *Cicer arietinum* | *CiarNCED1* | Ca.25800 | Phytozome v13 | v1.0 | Varshney et al. (2013) |
|  |  | *CiarNCED3* | Ca.05042 |  |  |  |
|  | *Citrus clementina* | *CclNCED3* | Ciclev10019364m_g_DQ309332_1 | Phytozome v13 | v1.0 | Wu et al. (2014) |
|  |  | *CclNCED5* | Cclementina_v1_0_Ciclev10014639m_g_DQ309329_1 |  |  |  |
|  |  | *CclNCED6* | Cclementina_v1_0_Ciclev10006710m_g_LOC18032346_XM_006420539 |  |  |  |
|  | *Citrus sinensis* | *CsNCED1* | orange1.1g007379m | Phytozome v13 | v1.1 | Wu et al. (2014) |
|  |  | *CsNCED2* | orange1.1g007291m |  |  |  |
|  |  | *CsNCED6* | orange1.1g044684m |  |  |  |
|  | *Coffea canephora* | *Cca3g7270* | - | Coffee Genome Hub | v1.0 | Denoeud et al. (2014) |
|  |  | *CcaNCED3* | Cca5g10210 |  |  |  |
|  | *Eucalyptus globulus* | *EglNCED1a* | CM024722.1 | GenBank Bioproject: PRJNA509734 | ASM1418254v1 | Australian National University |
|  |  | *EglNCED1b* | JABKBK010000006.1 |  |  |  |
|  |  | *EglNCED2* | EglChr4.NCED-1 |  |  |  |
|  |  | *EglNCED3* | EglChr4.NCED-2 |  |  |  |
|  | *Eucalyptus grandis* | *EgrNCED2a* | Eucgr.F01409 | Phytozome v13 | v2.0 | Myburg et al. (2014) |
|  | *Fragaria vesca* | *FveNCED1* | Fragaria_vesca_v4_0_a2_FvH4_4g05900_t1 | Phytozome v13 | v4.0.a2 | Li et al. (2019) |
|  |  | *FveNCED2* | Fragaria_vesca_v4_0_a2_FvH4_3g05440_t1 |  |  |  |
|  |  | *FveNCED3* | Fragaria_vesca_v4_0_a2_FvH4_3g16730_t1 |  |  |  |
|  | *Fragaria x ananassa* | *FaNCED1a* | Fx_ananassa_v1_0_a1_augustus_masked-Fvb4-2-processed-gene-211_5-mRNA-1 | Phytozome v13 | v1.0.a1 | Edger et al. (2019) |
|  |  | *FaNCED1b* | Fx_ananassa_v1_0_a1_augustus_masked-Fvb4-3-processed-gene-278_0-mRNA-1 |  |  |  |
|  |  | *FaNCED2a* | Fx_ananassa_v1_0_a1_augustus_masked-Fvb3-3-processed-gene-13_7-mRNA-1 |  |  |  |
|  |  | *FaNCED2b* | Fx_ananassa_v1_0_a1_augustus_masked-Fvb3-4-processed-gene-264_5-mRNA-1 |  |  |  |
|  |  | *FaNCED2c* | Fx_ananassa_v1_0_a1_snap_masked-Fvb3-2-processed-gene-7_21-mRNA-1 |  |  |  |
|  |  | *FaNCED2d* | Fx_ananassa_v1_0_a1_snap_masked-Fvb3-1-processed-gene-289_20-mRNA-1 |  |  |  |
|  |  | *FaNCED3a* | Fx_ananassa_v1_0_a1_augustus_masked-Fvb3-4-processed-gene-210_9-mRNA-1 |  |  |  |
|  |  | *FaNCED3b* | Fx_ananassa_v1_0_a1_augustus_masked-Fvb3-1-processed-gene-220_5-mRNA-1 |  |  |  |
|  |  | *FaNCED3c* | Fx_ananassa_v1_0_a1_augustus_masked-Fvb3-2-processed-gene-94_0-mRNA-1 |  |  |  |
|  |  | *FaNCED3d* | Fx_ananassa_v1_0_a1_augustus_masked-Fvb3-3-processed-gene-79_0-mRNA-1 |  |  |  |
|  | *Glycine max* | *GmNCED2* | Glyma.08G096200.1 | Phytozome v13 | Wm82.a4.v1 | Valliyodan et al. (2019) |
|  |  | *GmNCED3.1* | Glyma.05G140900.1 |  |  |  |
|  |  | *GmNCED3.2* | Glyma.08G176300.3 |  |  |  |
|  | *Gossypium arboreum* | *GaNCED2a* | Gar05G15470 | CottonGen | *Gossypium arboreum* (A2) 'SXY1' genome WHU-updated v1 | Yu et al. (2021) |
|  |  | *GaNCED2b* | Gar06G18530 |  |  |  |
|  |  | *GaNCED2c* | Gar10G04280 |  |  |  |
|  |  | *GaNCED3a* | Gar13G20540 |  |  |  |
|  |  | *GaNCED3b* | Gar08G30010 |  |  |  |
|  |  | *GaNCED3c* | Gar01G03850 |  |  |  |
|  |  | *GaNCED6* | Gar09G24600 |  |  |  |
|  | *Gossypium barbadense* | *GbNCED2aA* | Gobar.A05G154500.1 | Phytozome v13 | v1.1 | Chen et al. (2020) |
|  |  | *GbNCED2bA* | Gobar.A06G149200.1 |  |  |  |
|  |  | *GbNCED2cA* | Gobar.A10G045100.1 |  |  |  |
|  |  | *GbNCED2cD* | Gobar.D10G045300.1 |  |  |  |
|  |  | *GbNCED3aA* | Gobar.A13G168800.1 |  |  |  |
|  |  | *GbNCED3aD* | Gobar.D13G179400.1 |  |  |  |
|  |  | *GbNCED3bA* | Gobar.A08G251900.1 |  |  |  |
|  |  | *GbNCED3cA* | Gobar.A01G038100.1 |  |  |  |
|  |  | *GbNCED3cD* | Gobar.D01G035700.1 |  |  |  |
|  |  | *GbNCED6D* | Gobar.D09G216800.1 |  |  |  |
|  |  | *-* | Gobar.A09G213400.1 |  |  |  |
|  |  | *-* | Gobar.D05G153800.1 |  |  |  |
|  |  | *-* | Gobar.D08G279900.1 |  |  |  |
|  | *Gossypium hirsutum* | *GhNCED2aA* | Gohir.A05G148000.1 | Phytozome v13 | v2.1 | Chen et al. (2020) |
|  |  | *GhNCED2aD* | Gohir.D05G150500.1 |  |  |  |
|  |  | *GhNCED2bA* | Gohir.A06G137900.1 |  |  |  |
|  |  | *GhNCED2bD* | Gohir.D06G143100.1 |  |  |  |
|  |  | *GhNCED2cA* | Gohir.A10G041800.1 |  |  |  |
|  |  | *GhNCED3aA* | Gohir.A13G152200.1 |  |  |  |
|  |  | *GhNCED3aD* | Gohir.D13G156600.1 |  |  |  |
|  |  | *GhNCED3bA* | Gohir.A08G233700.1 |  |  |  |
|  |  | *GhNCED3bD* | Gohir.D08G253900.1 |  |  |  |
|  |  | *GhNCED3cA* | Gohir.A01G041300.1 |  |  |  |
|  |  | *GhNCED6A* | Gohir.A09G202400.1 |  |  |  |
|  |  | *GhNCED6D* | Gohir.D09G196400.1 |  |  |  |
|  | *Malus domestica* | *MdNCED1* | MD10G1194200 | Phytozome v13 | v1.1 | Volk et al. (2022) |
|  |  | *MdNCED2* | MD05G1207300 |  |  |  |
|  |  | *MdNCED3* | MD10G1261000 |  |  |  |
|  |  | *MdNCED4* | MD05G1282700 |  |  |  |
|  |  | *MdNCED4b* | MD16G1235500 |  |  |  |
|  | *Medicago truncatula* | *Medtr2g070460* | Medtr2g070460.1 | Phytozome v13 | Mt4.0v1 | Tang et al. (2014) |
|  | *Nicotiana attenuata* | *NatNCED1* | NIATv7.g16982.t1 | Sol Genomics Network | v7 | Xu et al. (2017) |
|  |  | *NatNCED2a* | NIATv7.g24598.t1 |  |  |  |
|  |  | *NatNCED2b* | NIATv7.g05302.t1 |  |  |  |
|  |  | *NatNCED3a* | NIATv7.g00854.t1 |  |  |  |
|  |  | *NatNCED3b* | NIATv7.g35209.t1 |  |  |  |
|  | *Nicotiana benthamiana* | *NbNCED1a* | Nbv0.5scaffold765 533346..531505 | QUT *Nicotiana benthamiana* Genome & Transcriptome (https://www.benthgenome.qut.edu.au/) | TrV6.1_LAB_Prm CDS/ Nbv05-genome-scaffolds | Naim et al. (2012); Kourelis et al. (2019) |
|  |  | *NbNCED1b* | Nbv0.5scaffold6061 116310..114475 |  |  |  |
|  |  | *NbNCED2* | Nbv6.1trP962/ Nbv0.5scaffold671 339475..337676 |  |  |  |
|  |  | *NbNCED3* | Nbv0.5scaffold3122 172651..174253 |  |  |  |
|  | *Nicotiana tabaccum* | *NtaNCED1* | Nitab4.5.0001924g0060.1 | Sol Genomics Network | v4.5 cds | Edwards et al. (2017) |
|  |  | *NtaNCED2a1* | Nitab4.5.0001070g0130.1 |  |  |  |
|  |  | *NtaNCED2a2* | Nitab4.5.0000420g0010.1 |  |  |  |
|  |  | *NtaNCED2b* | Nitab4.5.0000484g0060.1 |  |  |  |
|  |  | *NtaNCED3a* | Nitab4.5.0001066g0060.1 |  |  |  |
|  |  | *NtaNCED3b* | Nitab4.5.0001769g0030.1 |  |  |  |
|  | *Nicotiana tomentosiformis* | *NtoNCED2* | Ntom.mRNA.83277 | Sol Genomics Network | - | Sierro et al. (2013) |
|  | *Olea europaea* | *OeNCED1a* | Oeu050691 | Phytozome v13 | v1.0 | Unver et al. (2017) |
|  |  | *OeNCED1c* | Oeu058431 |  |  |  |
|  |  | *OeNCED2a* | Oeu034140 |  |  |  |
|  |  | *OeNCED2b* | Oeu052364 |  |  |  |
|  |  | *OeNCED2c* | Oeu040102 |  |  |  |
|  |  | *OeNCED2d* | Oeu042456 |  |  |  |
|  |  | *OeNCED3* | Oeu039694 |  |  |  |
|  | *Petunia axillaris* | *PaxNCED1* | Peaxi162Scf00111g00077.1 | Sol Genomics Network | v1.6.2 | Bombarely et al. (2016) |
|  |  | *PaxNCED2a* | Peaxi162Scf01211g10016.1 |  |  |  |
|  |  | *PaxNCED2b* | Peaxi162Scf00443g00001.1 |  |  |  |
|  | *Petunia inflata* | *PinNCED1* | Peinf101Scf00665g12001.1 | Sol Genomics Network | v1.0.1 | Bombarely et al. (2016) |
|  |  | *PinNCED2a* | Peinf101Scf01317g08004.1 |  |  |  |
|  |  | *PinNCED3* | Peinf101Scf00437g06002.1 |  |  |  |
|  | *Phaseolus vulgaris* | *PvNCED1* | Phvul.005G051600 | Phytozome v13 | v2.1 | DOE-JGI and USDA-NIFA, http://phytozome.jgi.doe.gov/ |
|  |  | *PvNCED3* | Phvul.007G198800 |  |  |  |
|  | *Pisum sativum* | *PsNCED2* | Psat1g001480 | URGI (plant and fungi data integration) | version 1a (cds) | Kreplak et al. (2019) |
|  |  | *PsNCED3* | Psat7g081800 |  |  |  |
|  | *Solanum lycopersicum* | *SlNCED1* | Solyc07g056570 | Phytozome v13 | ITAG4.0 | Hosmani et al. (2019) |
|  |  | *SlNCED2* | Solyc08g016720 |  |  |  |
|  |  | *SlNCED3* | Solyc05g053530 |  |  |  |
|  | *Solanum melongena* | *SmeNCED1* | SMEL4_07g020880.1 | Sol Genomics Network | v4.1 | Barchi et al. (2021) |
|  |  | *SmeNCED2* | SMEL4_08g019240.1 |  |  |  |
|  |  | *SmeNCED3* | SMEL4_05g002720.1 |  |  |  |
|  | *Solanum tuberosum* | *StNCED1* | AY662342.1 | Phytozome v13 | v4.03 | Xu et al. (2011) |
|  |  | *StNCED2* | AY662343.1 |  |  |  |
|  |  | *StNCED6* | LOC102579540 |  |  |  |
|  | *Theobroma cacao* | *TcNCED3* | Thecc.07G147400 | Phytozome v13 | v2.1 | Motamayor et al. (2013) |
|  |  | *TcNCED5* | Thecc.06G149100 |  |  |  |
|  |  | *TcNCED6* | Thecc.04G210600 |  |  |  |
|  | *Vigna unguiculata* | *VuNCED1* | Vigun01g052600 | Phytozome v13 | v1.2 | Lonardi et al. (2019) |
|  |  | *VuNCED3* | Vigun07g142600 |  |  |  |
|  | *Vitis vinifera* | *VvNCED1* | VIT.219s0093g00550 | Phytozome v13 | v2.1 | Jaillon et al. (2007) |
|  |  | *VvNCED2* | VIT.210s0003g03750 |  |  |  |
|  |  | *VvNCED3* | VIT.205s0051g00670 |  |  |  |

**Supplementary References:**

Albert VA, Barbazuk WB, DePamphilis CW, et al (2013) The *Amborella* genome and the evolution of flowering plants. Science 342:1241089. https://doi.org/10.1126/science.1241089

Banks JA, Nishiyama T, Hasebe M, et al (2011) The Selaginella genome identifies genetic changes associated with the evolution of vascular plants. Science 332:960–963. https://doi.org/10.1126/science.1203810

Barchi L, Rabanus-Wallace MT, Prohens J, et al (2021) Improved genome assembly and pan-genome provide key insights into eggplant domestication and breeding. Plant J 107:579–596. https://doi.org/10.1111/tpj.15313

Barrero JM, Rodríguez PL, Quesada V, et al (2006) Both abscisic acid (ABA)-dependent and ABA-independent pathways govern the induction of *NCED3*, *AAO3* and *ABA1* in response to salt stress. Plant Cell Environ 29:2000–2008. https://doi.org/10.1111/j.1365-3040.2006.01576.x

Beier S, Himmelbach A, Colmsee C, et al (2017) Construction of a map-based reference genome sequence for barley, *Hordeum vulgare* L. Sci Data 4:170044. https://doi.org/10.1038/sdata.2017.44

Bertioli DJ, Jenkins J, Clevenger J, et al (2019) The genome sequence of segmental allotetraploid peanut *Arachis hypogaea*. Nat Genet 51:877–884. https://doi.org/10.1038/s41588-019-0405-z

Bombarely A, Moser M, Amrad A, et al (2016) Insight into the evolution of the Solanaceae from the parental genomes of *Petunia hybrida*. Nat Plants 2:16074. https://doi.org/10.1038/nplants.2016.74

Bowman JL, Kohchi T, Yamato KT, et al (2017) Insights into land plant evolution garnered from the *Marchantia polymorpha* genome. Cell 171:287-304.e15. https://doi.org/10.1016/j.cell.2017.09.030

Chen ZJ, Sreedasyam A, Ando A, et al (2020) Genomic diversifications of five *Gossypium* allopolyploid species and their impact on cotton improvement. Nat Genet 52:525–533. https://doi.org/10.1038/s41588-020-0614-5

Cheng C-Y, Krishnakumar V, Chan AP, et al (2017) Araport11: a complete reannotation of the *Arabidopsis thaliana* reference genome. Plant J 89:789–804. https://doi.org/10.1111/tpj.13415

Clark K, Karsch-Mizrachi I, Lipman DJ, et al (2016) GenBank. Nucleic Acids Res 44:D67-72. https://doi.org/10.1093/nar/gkv1276

Czechowski T, Stitt M, Altmann T, Udvardi MK (2005) Genome-wide identification and testing of superior reference genes for transcript normalization in Arabidopsis. Plant Physiol 139:5–17. https://doi.org/10.1104/pp.105.063743.1

D’Hont A, Denoeud F, Aury J-M, et al (2012) The banana (*Musa acuminata*) genome and the evolution of monocotyledonous plants. Nature 488:213–217. https://doi.org/10.1038/nature11241

Denoeud F, Carretero-Paulet L, Dereeper A, et al (2014) The coffee genome provides insight into the convergent evolution of caffeine biosynthesis. Science 345:1181–1184. https://doi.org/10.1126/science.1255274

Die JV, Román B, Nadal S, González-Verdejo CI (2010) Evaluation of candidate reference genes for expression studies in *Pisum sativum* under different experimental conditions. Planta 232:145–153. https://doi.org/10.1007/s00425-010-1158-1

Dunnett CW (1964) New tables for multiple comparisons with a control. Biometrics 20:482–491

Edger PP, Poorten TJ, VanBuren R, et al (2019) Origin and evolution of the octoploid strawberry genome. Nat Genet 51:541–547. https://doi.org/10.1038/s41588-019-0356-4

Edwards KD, Fernandez-Pozo N, Drake-Stowe K, et al (2017) A reference genome for *Nicotiana tabacum* enables map-based cloning of homeologous loci implicated in nitrogen utilization efficiency. BMC Genomics 18:448. https://doi.org/10.1186/s12864-017-3791-6

Falk T, Herndon N, Grau E, et al (2018) Growing and cultivating the forest genomics database, TreeGenes. Database (Oxford) 2018:1–11. https://doi.org/10.1093/database/bay084

Fernandez-Pozo N, Menda N, Edwards JD, et al (2015) The Sol Genomics Network (SGN)--from genotype to phenotype to breeding. Nucleic Acids Res 43:D1036-D1041. https://doi.org/10.1093/nar/gku1195

Filiault DL, Ballerini ES, Mandáková T, et al (2018) The *Aquilegia* genome provides insight into adaptive radiation and reveals an extraordinarily polymorphic chromosome with a unique history. Elife 7:e36426. https://doi.org/10.7554/eLife.36426

Garsmeur O, Droc G, Antonise R, et al (2018) A mosaic monoploid reference sequence for the highly complex genome of sugarcane. Nat Commun 9:2638. https://doi.org/10.1038/s41467-018-05051-5

Gernhard T (2008) The conditioned reconstructed process. J Theor Biol 253:769–778. https://doi.org/10.1016/j.jtbi.2008.04.005

Goodstein DM, Shu S, Howson R, et al (2012) Phytozome: a comparative platform for green plant genomics. Nucleic Acids Res 40:D1178–D1186. https://doi.org/10.1093/nar/gkr944

Hosmani PS, Flores-Gonzalez M, van de Geest H, et al (2019) An improved de novo assembly and annotation of the tomato reference genome using single-molecule sequencing, Hi-C proximity ligation and optical maps. bioRxiv 767764. https://doi.org/10.1101/767764

Jaillon O, Aury J-M, Noel B, et al (2007) The grapevine genome sequence suggests ancestral hexaploidization in major angiosperm phyla. Nature 449:463–467. https://doi.org/10.1038/nature06148

Jarvis DE, Ho YS, Lightfoot DJ, et al (2017) The genome of *Chenopodium quinoa*. Nature 542:307–312. https://doi.org/10.1038/nature21370

Katoh K, Misawa K, Kuma K, Miyata T (2002) MAFFT: a novel method for rapid multiple sequence alignment based on fast Fourier transform. Nucleic Acids Res 30:3059–3066. https://doi.org/10.1093/nar/gkf436

Katoh K, Standley DM (2013) MAFFT Multiple Sequence Alignment Software Version 7: Improvements in performance and usability. Mol Biol Evol 30:772–780. https://doi.org/10.1093/molbev/mst010

Kim S, Park M, Yeom SI, et al (2014) Genome sequence of the hot pepper provides insights into the evolution of pungency in *Capsicum* species. Nat Genet 46:270–278. https://doi.org/10.1038/ng.2877

Kourelis J, Kaschani F, Grosse-Holz FM, et al (2019) A homology-guided, genome-based proteome for improved proteomics in the alloploid *Nicotiana benthamiana*. BMC Genomics 20:722. https://doi.org/10.1186/s12864-019-6058-6

Kreplak J, Madoui M-A, Cápal P, et al (2019) A reference genome for pea provides insight into legume genome evolution. Nat Genet 51:1411–1422. https://doi.org/10.1038/s41588-019-0480-1

Lanfear R, Calcott B, Ho SYW, Guindon S (2012) PartitionFinder: Combined selection of partitioning schemes and substitution models for phylogenetic analyses. Mol Biol Evol 29:1695–1701. https://doi.org/10.1093/molbev/mss020

Lanfear R, Frandsen PB, Wright AM, et al (2017) PartitionFinder 2: New methods for selecting partitioned models of evolution for molecular and morphological phylogenetic analyses. Mol Biol Evol 34:772–773. https://doi.org/10.1093/molbev/msw260

Lang D, Ullrich KK, Murat F, et al (2018) The *Physcomitrella patens* chromosome-scale assembly reveals moss genome structure and evolution. Plant J 93:515–533. https://doi.org/10.1111/tpj.13801

Lee H, Chawla HS, Obermeier C, et al (2020) Chromosome-scale assembly of winter oilseed rape *Brassica napus*. Front Plant Sci 11:496

Leebens-Mack JH, Barker MS, Carpenter EJ, et al (2019) One thousand plant transcriptomes and the phylogenomics of green plants. Nature 574:679–685. https://doi.org/10.1038/s41586-019-1693-2

Li F-W, Brouwer P, Carretero-Paulet L, et al (2018) Fern genomes elucidate land plant evolution and cyanobacterial symbioses. Nat Plants 4:460–472. https://doi.org/10.1038/s41477-018-0188-8

Li F-W, Nishiyama T, Waller M, et al (2020) *Anthoceros* genomes illuminate the origin of land plants and the unique biology of hornworts. Nat Plants 6:259–272. https://doi.org/10.1038/s41477-020-0618-2

Li Y, Pi M, Gao Q, et al (2019) Updated annotation of the wild strawberry *Fragaria vesca* V4 genome. Hortic Res 6:61. https://doi.org/10.1038/s41438-019-0142-6

Liu H, Wang X, Wang G, et al (2021) The nearly complete genome of *Ginkgo biloba* illuminates gymnosperm evolution. Nat Plants 7:748–756. https://doi.org/10.1038/s41477-021-00933-x

Liu S, Liu Y, Yang X, et al (2014) The *Brassica oleracea* genome reveals the asymmetrical evolution of polyploid genomes. Nat Commun 5:3930. https://doi.org/10.1038/ncomms4930

Lonardi S, Muñoz-Amatriaín M, Liang Q, et al (2019) The genome of cowpea (*Vigna unguiculata* [L.] Walp.). Plant J 98:767–782. https://doi.org/10.1111/tpj.14349

Lyons E, Freeling M (2008) How to usefully compare homologous plant genes and chromosomes as DNA sequences. Plant J 53:661–673. https://doi.org/10.1111/j.1365-313X.2007.03326.x

Lyons E, Pedersen B, Kane J, et al (2008) Finding and comparing syntenic regions among Arabidopsis and the outgroups papaya, poplar, and grape: CoGe with Rosids. Plant Physiol 148:1772–1781. https://doi.org/10.1104/pp.108.124867

Marchant DB, Chen G, Cai S, et al (2022) Dynamic genome evolution in a model fern. Nat Plants 8:1038–1051. https://doi.org/10.1038/s41477-022-01226-7

Mayer KFX, Rogers J, Doležel J, et al (2014) A chromosome-based draft sequence of the hexaploid bread wheat (*Triticum aestivum*) genome. Science 345:1251788. https://doi.org/10.1126/science.1251788

McAdam SAM, Sussmilch FC, Brodribb TJ (2016) Stomatal responses to vapour pressure deficit are regulated by high speed gene expression in angiosperms. Plant Cell Environ 39:485–491. https://doi.org/10.1111/pce.12633

McCormick RF, Truong SK, Sreedasyam A, et al (2018) The *Sorghum bicolor* reference genome: Improved assembly, gene annotations, a transcriptome atlas, and signatures of genome organization. Plant J 93:338–354. https://doi.org/10.1111/tpj.13781

McGrath JM, Funk A, Galewski P, et al (2022) A contiguous de novo genome assembly of sugar beet EL10 (*Beta vulgaris* L.). DNA Res 30:dsac033. https://doi.org/10.1093/dnares/dsac033

Ming R, VanBuren R, Wai CM, et al (2015) The pineapple genome and the evolution of CAM photosynthesis. Nat Genet 47:1435–1442. https://doi.org/10.1038/ng.3435

Motamayor JC, Mockaitis K, Schmutz J, et al (2013) The genome sequence of the most widely cultivated cacao type and its use to identify candidate genes regulating pod color. Genome Biol 14:r53. https://doi.org/10.1186/gb-2013-14-6-r53

Myburg AA, Grattapaglia D, Tuskan GA, et al (2014) The genome of *Eucalyptus grandis*. Nature 510:356–362. https://doi.org/10.1038/nature13308

Naim F, Nakasugi K, Crowhurst RN, et al (2012) Advanced engineering of lipid metabolism in *Nicotiana benthamiana* using a draft genome and the V2 viral silencing-suppressor protein. PLoS One 7:e52717

Nystedt B, Street NR, Wetterbom A, et al (2013) The Norway spruce genome sequence and conifer genome evolution. Nature 497:579–584. https://doi.org/10.1038/nature12211

Ouyang S, Zhu W, Hamilton J, et al (2007) The TIGR Rice Genome Annotation Resource: improvements and new features. Nucleic Acids Res 35:D883–D887. https://doi.org/10.1093/nar/gkl976

Petruzzellis F, Savi T, Bacaro G, Nardini A (2019) A simplified framework for fast and reliable measurement of leaf turgor loss point. Plant Physiol Biochem 139:395–399. https://doi.org/10.1016/j.plaphy.2019.03.043

Rendón-Anaya M, Ibarra-Laclette E, Méndez Bravo A, et al (2019) The avocado genome informs deep angiosperm phylogeny, highlights introgressive hybridization, and reveals pathogen-influenced gene space adaptation. Proc Natl Acad Sci USA 116:17081–17089. https://doi.org/10.1101/654285

Rodriguez-Dominguez CM, Forner A, Martorell S, et al (2022) Leaf water potential measurements using the pressure chamber: Synthetic testing of assumptions towards best practices for precision and accuracy. Plant Cell Environ 45:2037–2061. https://doi.org/10.1111/pce.14330

Sayers EW, Bolton EE, Brister JR, et al (2022) Database resources of the National Center for Biotechnology Information. Nucleic Acids Res 50:D20–D26. https://doi.org/10.1093/nar/gkab1112

Schnable PS, Ware D, Fulton RS, et al (2009) The B73 maize genome: complexity, diversity, and dynamics. Science 326:1112–1115. https://doi.org/10.1126/science.1178534

Sierro N, Battey JND, Ouadi S, et al (2013) Reference genomes and transcriptomes of *Nicotiana sylvestris* and *Nicotiana tomentosiformis*. Genome Biol 14:R60. https://doi.org/10.1186/gb-2013-14-6-r60

Suchard MA, Lemey P, Baele G, et al (2018) Bayesian phylogenetic and phylodynamic data integration using BEAST 1.10. Virus Evol 4:vey016. https://doi.org/10.1093/ve/vey016

Sundell D, Mannapperuma C, Netotea S, et al (2015) The Plant Genome Integrative Explorer Resource: PlantGenIE.org. New Phytol 208:1149–1156. https://doi.org/10.1111/nph.13557

Tan BC, Joseph LM, Deng WT, et al (2003) Molecular characterisation of the Arabidopsis 9-*cis*-epoxycarotenoid dioxygenase gene family. Plant J 35:44–56. https://doi.org/10.1046/j.1365-313X.2003.01786.x

Tang H, Krishnakumar V, Bidwell S, et al (2014) An improved genome release (version Mt4.0) for the model legume *Medicago truncatula*. BMC Genomics 15:312. https://doi.org/10.1186/1471-2164-15-312

Unver T, Wu Z, Sterck L, et al (2017) Genome of wild olive and the evolution of oil biosynthesis. Proc Natl Acad Sci USA 114:E9413–E9422. https://doi.org/10.1073/pnas.1708621114

Valliyodan B, Cannon SB, Bayer PE, et al (2019) Construction and comparison of three reference-quality genome assemblies for soybean. Plant J 100:1066–1082. https://doi.org/10.1111/tpj.14500

Varshney RK, Song C, Saxena RK, et al (2013) Draft genome sequence of chickpea (*Cicer arietinum*) provides a resource for trait improvement. Nat Biotechnol 31:240–246. https://doi.org/10.1038/nbt.2491

Volk GM, Peace CP, Henk AD, Howard NP (2022) DNA profiling with the 20K apple SNP array reveals *Malus domestica* hybridization and admixture in *M. sieversii, M. orientalis*, and *M. sylvestris* genebank accessions. Front Plant Sci 13:1015658. https://doi.org/10.3389/fpls.2022.1015658

Wan T, Liu Z-M, Li L-F, et al (2018) A genome for gnetophytes and early evolution of seed plants. Nat Plants 4:82–89. https://doi.org/10.1038/s41477-017-0097-2

Wegrzyn JL, Lee JM, Tearse BR, Neale DB (2008) TreeGenes: A forest tree genome database. Int J Plant Genomics 2008:412875. https://doi.org/10.1155/2008/412875

Wegrzyn JL, Staton MA, Street NR, et al (2019) Cyberinfrastructure to improve forest health and productivity: The role of tree databases in connecting genomes, phenomes, and the environment. Front Plant Sci 10:813

Wickell D, Kuo L-Y, Yang H-P, et al (2021) Underwater CAM photosynthesis elucidated by Isoetes genome. Nat Commun 12:6348. https://doi.org/10.1038/s41467-021-26644-7

Wu GA, Prochnik S, Jenkins J, et al (2014) Sequencing of diverse mandarin, pummelo and orange genomes reveals complex history of admixture during citrus domestication. Nat Biotechnol 32:656–662. https://doi.org/10.1038/nbt.2906

Xu S, Brockmöller T, Navarro-Quezada A, et al (2017) Wild tobacco genomes reveal the evolution of nicotine biosynthesis. Proc Natl Acad Sci USA114:6133–6138. https://doi.org/10.1073/pnas.1700073114

Xu X, Pan S, Cheng S, et al (2011) Genome sequence and analysis of the tuber crop potato. Nature 475:189–195. https://doi.org/10.1038/nature10158

Yu J, Jung S, Cheng C-H, et al (2021) CottonGen: The community database for cotton genomics, genetics, and breeding research. Plants 10:2805. https://doi.org/10.3390/plants10122805

Zhang FP, Sussmilch F, Nichols DS, et al (2018a) Leaves, not roots or floral tissue, are the main site of rapid, external pressure-induced ABA biosynthesis in angiosperms. J Exp Bot 69:1261–1267. https://doi.org/10.1093/jxb/erx480

Zhang L, Cai X, Wu J, et al (2018b) Improved *Brassica rapa* reference genome by single-molecule sequencing and chromosome conformation capture technologies. Hortic Res 5:50. https://doi.org/10.1038/s41438-018-0071-9
